# 3pHLA-score improves structure-based peptide-HLA binding affinity prediction

**DOI:** 10.1038/s41598-022-14526-x

**Published:** 2022-06-24

**Authors:** Anja Conev, Didier Devaurs, Mauricio Menegatti Rigo, Dinler Amaral Antunes, Lydia E. Kavraki

**Affiliations:** 1 Department of Computer Science, Rice University, Houston, 77005 USA; 2 MRC Institute of Genetics and Cancer, University of Edinburgh, Edinburgh, EH4 2XU UK; 3 Department of Biology and Biochemistry, University of Houston, Houston, 77004 USA

**Keywords:** Computational biology and bioinformatics, Computational models, Machine learning, MHC, Peptide vaccines

## Abstract

Binding of peptides to Human Leukocyte Antigen (HLA) receptors is a prerequisite for triggering immune response. Estimating peptide-HLA (pHLA) binding is crucial for peptide vaccine target identification and epitope discovery pipelines. Computational methods for binding affinity prediction can accelerate these pipelines. Currently, most of those computational methods rely exclusively on sequence-based data, which leads to inherent limitations. Recent studies have shown that structure-based data can address some of these limitations. In this work we propose a novel machine learning (ML) structure-based protocol to predict binding affinity of peptides to HLA receptors. For that, we engineer the input features for ML models by decoupling energy contributions at different residue positions in peptides, which leads to our novel per-peptide-position protocol. Using Rosetta’s ref2015 scoring function as a baseline we use this protocol to develop 3pHLA-score. Our per-peptide-position protocol outperforms the standard training protocol and leads to an increase from 0.82 to 0.99 of the area under the precision-recall curve. 3pHLA-score outperforms widely used scoring functions (AutoDock4, Vina, Dope, Vinardo, FoldX, GradDock) in a structural virtual screening task. Overall, this work brings structure-based methods one step closer to epitope discovery pipelines and could help advance the development of cancer and viral vaccines.

## Introduction

Human Leukocite Antigen (HLA) class I molecules are an important part of human cellular immune response^1,2^. HLAs are involved in the intracellular antigen presentation pathway; they are responsible for the transport and display of peptide antigens for T-cell scrutiny^3,4^. Therefore, the possibility of exploiting the HLA role in this pathway to engineer immune responses has shown great promise^5^, as highlighted by efforts on personalized peptide vaccine development^6^. When designing peptide vaccines, a pool of potential peptide targets is identified from a protein of interest. Targets are then filtered to identify those most likely to induce an immune response. This whole process is referred to as epitope discovery^7^. Discovered immunogenic epitopes are able to bind HLA receptors, create stable peptide-HLA (pHLA) complexes (Fig. S1) and induce an immunological response^8^. Unfortunately, epitope discovery is made challenging by the high diversity of HLA molecules. This diversity is a reflection of the high number of HLA alleles: more than 24,000 HLA-I alleles have been identified to date^9^. Each allele codes for a specific HLA receptor (e.g., HLA-A0201, HLA-B0702) with different peptide binding preferences. Fast and accurate computational evaluation of pHLA binding can speed up the search for epitopes and is an important part of epitope discovery pipelines.

So far computational pHLA binding affinity prediction efforts have been largely dominated by sequence-based approaches^10–15,58^. While these methods provide good accuracy and are a part of many existing pipelines, they have some inherent drawbacks^16^. For instance, they rely on a predefined amino acid alphabet to represent the pHLA. Most existing tools have canonical amino acids in their alphabet^10–12^ and are thus unable to process phosphorylated peptides, although these peptides can be displayed by HLAs^17^. While recent efforts^18^ expand the alphabet to include phosphorylation, the problem of the predefined alphabet persists. The presence of other post-translational modifications or small molecules within the binding site cannot be taken into account by such approaches. In addition, sequence-based predictors are highly dependent on the quality and composition of the training set^19,20^. This represents an important limitation because of the aforementioned high diversity of HLA alleles^21^. All these challenges indicate that sequence-based methods alone can not identify all relevant epitopes, which motivates further exploration and development of complementary approaches^22^.

Structure-based methods use three-dimensional arrangements (i.e., conformations) of receptors and ligands^23^. They are not restricted to a predefined amino acid alphabet and can be used in docking or structural virtual screening tasks^24^. In the context of these tasks, structure-based scoring functions are used to approximate the free energy of a molecular system. Most scoring functions are generic and can be used to score any complex of interest (including pHLAs), but their performance is often system-dependent^25^. To tailor scoring functions to a specific protein family, machine learning (ML) efforts are emerging^26,27^. As reliable pHLA modeling tools arise^23,28^, and more data become available, we see a potential for pHLA ML scoring functions and structure-based methods to enter epitope discovery pipelines and complement existing sequence-based methods.

Under the hood, most scoring functions (such as Rosetta’s ref2015^29^) approximate independent energy terms for a molecular complex and rely on the assumption that binding affinity can be described as a weighted sum of these terms^30^. Standard ML training protocols use the same assumption. GradDock^31^, for example, involves ref2015 standard energy terms and redefines their weights to better fit the HLA system while keeping the additive formulation. However, this additive functional form of classical (and ML-derived) scoring functions has been challenged in previous studies^32,33^. SIEVE-Score^34^ recently considered binding site residues and exemplified the benefit of decomposing the energy terms associated with binding site residues for interaction-energy-based learning. The idea of assessing peptide binding affinity via decomposition into peptide residues has also been applied in the context of other computational approaches with mixed results^35^, such as quantitative structure-activity relationship (QSAR) studies involving amino acid descriptors^36^.

In our approach, we decompose the energy terms of a pHLA complex into separate contributions for all residues at each position in the peptide; we then use these energy terms as input to train ML models for binding affinity prediction. We call this approach the per-peptide-position training protocol. Our rationale is that structural information that is important for pHLA binding prediction gets lost when standard scoring functions (involving the additive formulation) are applied to the pHLA complex. We use our per-peptide-position protocol in the context of the Rosetta framework^37^, which leads to our novel 3pHLA-score. The main novelty of our work resides in the combination of two complementary ideas in an innovative fashion: (1) tuning the weights of Rosetta’s scoring function to more accurately assess pHLA binding; (2) keeping the energy terms associated with peptide’s residue positions separate to not lose information through aggregation.

We evaluate the predictive power of our per-peptide-position protocol in the first set of experiments where we compare **3pHLA-score** with the baseline **ref2015-score** and the **standard-HLA-score** trained using the standard additive protocol. Our results show a clear lead of the per-peptide-position protocol over the standard training protocol and the default ref2015 scoring function. We then validate 3pHLA-score on two independent datasets and compare it to six widely used scoring functions: AutoDock4^38^, Vina^39^, Vinardo^40^, GradDock^31^, DOPE^41^, FoldX^42^. 3pHLA-score outperforms the other scoring function in the virtual screening setting and shows the ability to generalize well on the independent datasets. This work provides a guideline for future development of ML structure-based scoring functions. Furthermore, it brings structure-based methods closer to epitope discovery pipelines, which could help advance the development of peptide vaccines.

## Methods

In this work, we train ML models on pHLA energy terms that are decomposed into specific contributions associated with each residue position within a peptide. We call this approach the per-peptide-position protocol and we apply it to Rosetta’s ref2015 energy terms to build our 3pHLA-score. Hence, in order to explain our work, we need to first describe the ref2015-score. In addition, we describe a score that we call standard-pHLA-score which uses an intermediate protocol between ref2015 and 3pHLA-score, as it is trained for the pHLA system using the original ref2015 energy terms without decomposition.

### Baseline ref2015-score

**Figure 1:**
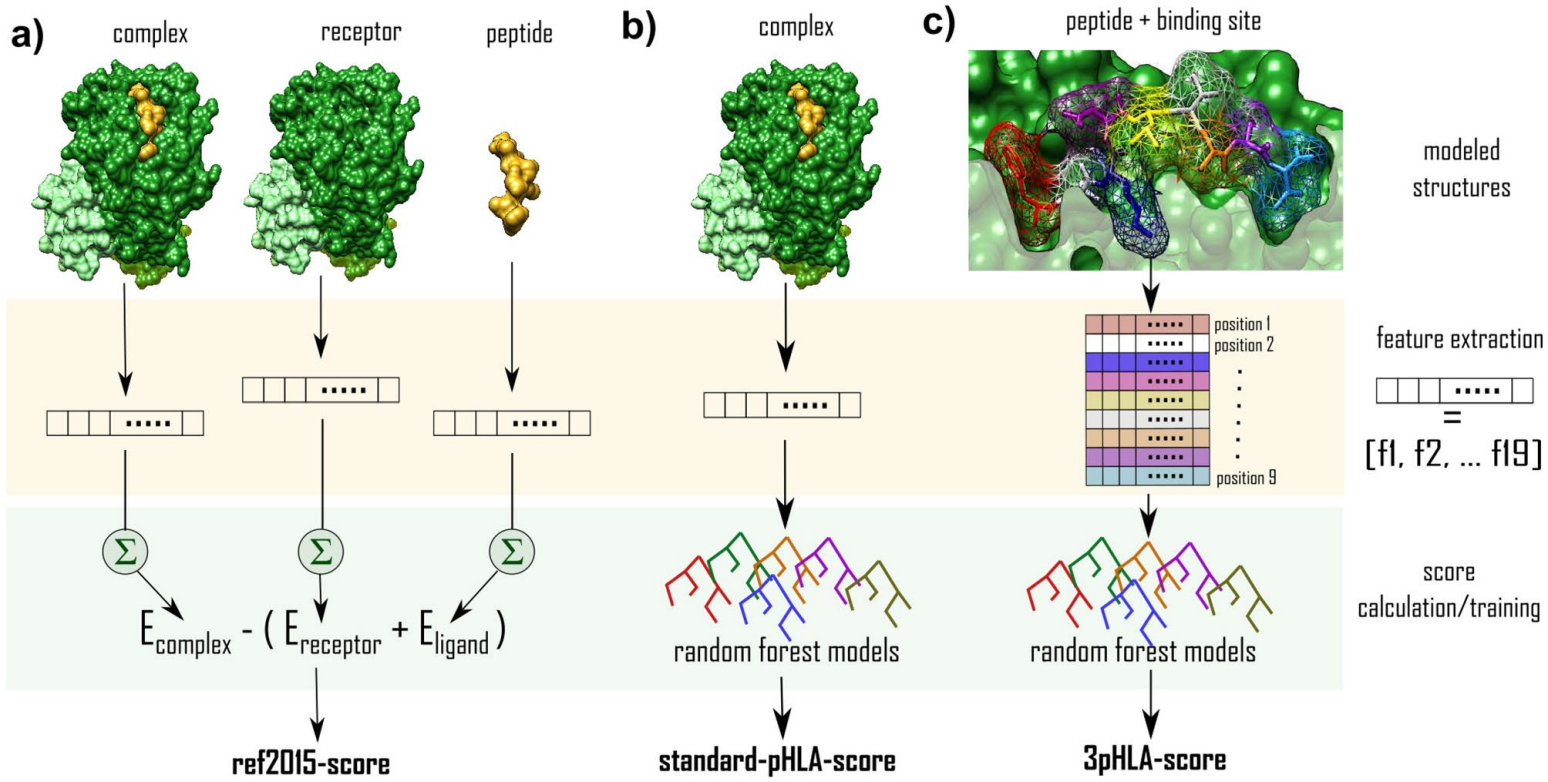
Description of three different protocols for approximating the binding affinity of a pHLA complex. Example input structures are visualized in the first row. The second row (orange stripe) shows the feature extraction phase of the scoring where ref2015 energy terms are extracted (Supplementary Table S1). The score calculation and training phase are indicated in row 3 (green stripe). **(a)** For ref2015-score, standard ref2015 energies are calculated for the complex, receptor, and ligand. They are then used to derive the binding energy with the Eq. (1) **(b)** For standard-pHLA-score, standard features are extracted from the complex; scoring is done using trained random forest regression models. **(c)** For the 3pHLA-score, per-peptide-position features are extracted from the structure of the complex; scoring is done using trained random forest regression models.

The 3D conformation of a given pHLA complex is stored in a PDB (Protein DataBank^43^) file containing the coordinates of all the atoms in this molecular complex (Fig. 1a). Rosetta’s ref2015 scoring function feeds this all-atom information into pre-parametrized mathematical and physical models to calculate different energy terms^29^. These energy terms are based on predefined equations that model different chemical and physical aspects of a molecular system, such as electrostatics, hydrogen bonding, and van der Waals interactions. The ref2015 scoring function contains 19 energy terms listed in Supplementary Table S1. The total energy of the input structure is approximated as a linear weighted sum of these energy terms. The default weights of ref2015 have been optimized on a wide range of scientific benchmarks to bring Rosetta calculations in agreement with small-molecule thermodynamic data and high-resolution structural features^29^. In this study, we approximate binding energy using ref2015-score with the equation^44^:

1 $$\begin{aligned} E_{binding} = E_{complex} - (E_{receptor}+E_{peptide}) \end{aligned}$$

where $E_{complex}$ is the ref2015 energy of the whole complex, $E_{receptor}$ is the ref2015 energy of the HLA receptor alone and $E_{peptide}$ is the ref2015 energy of the peptide (Fig. 1a).

### Standard-pHLA-score

ML models can be used to refine scoring functions and tailor them to a specific system of interest. However, they do not have priors on physical and chemical properties of the molecular system. If all-atom coordinates are used as features, they can introduce noise which slows down the training and makes the learning process more difficult. This is why an initial step of transforming the structural information into compact features is needed. A standard protocol is to use the energy terms provided by traditional scoring functions as features (i.e., inputs to the models) and tune their weights to fit a particular system^31,45^. We formulate the standard ref2015 features as a vector containing the 19 ref2015 energy terms. We train non-linear ML models (see Machine learning models subsection) using these standard features to develop the standard-pHLA-score (Fig. 1b).

### 3pHLA-score

To develop 3pHLA-score, we go beyond the standard featurization. We decompose ref2015 energy terms into energy contributions associated with each residue position in the peptide, which we call per-peptide-position features. This protocol is inspired by the domain knowledge about the pHLA complex. Experimental findings on peptide anchors suggest that important information about the binding can be retrieved by zooming into the energy of the binding pocket at specific regions surrounding different positions in the peptide^46^. To extract the per-peptide-position features, we first scored the whole pHLA complex with Rosetta’s ref2015 (as explained in the subsection above). Next, we applied PyRosetta’s^47^ *residue_total_energies_array* function. This function allows us to see how the structural energy of the complex breaks down into per-peptide-position contributions. The output of *residue_total_energies_array* is an array of energy terms (Table S1) for each peptide residue position, which we stack to form the input vector (see Supplementary Material subsection *Per-peptide-position feature vector*). This vector is used as input to the non-linear ML models (see Machine learning models subsection) to create 3pHLA-score (Fig. 1c).

### Machine learning models

For standard-pHLA-score and 3pHLA-score we used the same dataset and settings to train our ML models - they only differ in the input features extracted from molecular structures.

We trained Random Forest Regression models^48^ on a per-HLA-allele basis. For each featurization, we trained 28 models - one for each HLA allele in the dataset. We built regression trees using the CART algorithm^49^ with the mean absolute error as the split criterion. To create ensembles of regression trees we used bootstrap aggregation. We scaled experimental binding affinities into the [0,1] range^11,12^ (Eq. S.3) and used them as prediction targets.

We compiled the training set by extracting 90% of binders and 90% of non-binders with equally distributed binding affinities out of Dataset 1 (see below). The rest of the data constitutes the test set, which was left out of the training and cross-validation phase. We stratified the training set into 5 folds (each with equal distribution of binding affinities) for hyperparameter tuning in a 5-fold cross-validation setting. Using randomized search and the 5-fold cross-validation we tuned the following parameters: number of trees, number of features per tree, maximum tree depth, and minimum samples per leaf. After tuning, we evaluated the performance of the final models on the left-out test set.

Note that our main experiments describe the use of Random Forest Regression models for training the standard-pHLA-score and 3pHLA-score. However, we assessed other regression techniques: linear regression, support vector machine regression, and partial least squares regression. We provide related results and discussion in the *Alternative ML regression techniques* subsection of the Supplementary Material.

### Dataset 1

This dataset consists of 77,581 pHLA structures modeled by the APE-Gen modeling tool^28,50^. It involves 28 HLA alleles (13 HLA-A, 12 HLA-B, and 3 HLA-C alleles). Peptides included in this dataset are all of the length 9 (9-mers). The experimental binding affinity of each pHLA complex was extracted from MHCFlurry^10^, which used IEDB^51^ as its main source of information. As mentioned above, Dataset 1 was split into non-overlapping training and test portions to separately train and evaluate 3pHLA-score and standard-ref2015-score.

### Dataset 2

Dataset 2 is an evaluation dataset containing 100 strong binders experimentally identified and curated in related work^10^ along with 2000 additional pHLA decoys extracted from the NetMHC dataset^11^. Selected pHLA complexes have no overlap with the training set (which is a subset of Dataset 1) and were modeled with APE-Gen using the methodology proposed in the reference study^28^. Dataset 2 was composed to mimic an epitope discovery setting where a large pool of peptide targets is screened, but only a small portion of the targets are true binders.

### Dataset 3

Dataset 3 is an evaluation set containing 11 pHLA complexes for the HLA-A0201 allele with different levels of known experimental binding affinity (strong [0-5] nM, medium [50-500] nM and weak [500-25,000] nM) for which there exist crystal structures in the PDB. Three out of 11 peptides are 10-mers while the others are 9-mers. We collected crystal structures for each of the pHLA complexes (note that there were multiple entries for some complex complexes, see Supplementary Table S4). Multiple biological assemblies sometimes with alternative side chain positions were extracted from each PDB file and treated as separate structures. This led to the inclusion of 77 structures in Dataset 3. Preprocessing of the crystals was done using PyMol^52^ (to remove water molecules and hydrogen atoms) and pdbfixer^53^ (to add missing atoms). Since crystal structures of complexes involving non-binder peptides do not exist, five additional structures of experimentally determined non-binding peptides^50^ for the HLA-A0201 allele were modeled with Docktope^54^ and added to Dataset 3. The complete dataset is outlined in Supplementary Table S4; it contains 82 structures of pHLA complexes involving 16 peptides and the HLA-A0201 receptor. These pHLA complexes do not appear in the training set (which is a subset of Dataset 1). Dataset 3 is a good test of the generalizability of 3pHLA-score because it strongly differs from the training dataset - structures are not modeled by APE-Gen and some involve peptides of length 10.

### Comparison of scoring functions

Several evaluation metrics were used to compare the performance of scoring functions (see Supplementary Material section Evaluation Metrics). Because we focused on assessing how well the functions could reproduce peptide rankings in terms of HLA-binding affinity, we used Pearson’s correlation coefficient *r* and Spearman’s correlation coefficient $\rho$ to evaluate the regression performance. To assess classification power, we used the Area Under the Receiver Operator Curve (AUROC) and the Area Under the Precision-Recall Curve (AUPRC). The performance of 3pHLA-score on Dataset 2 and Dataset 3 was compared to other widely used scoring functions which use different techniques (Table S5). When visualized, scores were scaled using max normalization to fit [0-1] range, but inverted such that values closer to 1 represent stronger binders for all investigated scoring functions, while values closer to 0 represent weaker binders.

### Human and animal rights

No human or animal data samples were used in this study.

## Results

We investigate the benefits of our per-peptide-position protocol by assessing the predictive power of 3pHLA-score on the test portion of Dataset 1 (see Methods subsection Dataset 1). We then compare the performance of the 3pHLA-score to six other widely used scoring functions in two different settings using independent datasets: Dataset 2 and Dataset 3.

### Per-peptide-position featurization shows superior predictive power

**Figure 2:**
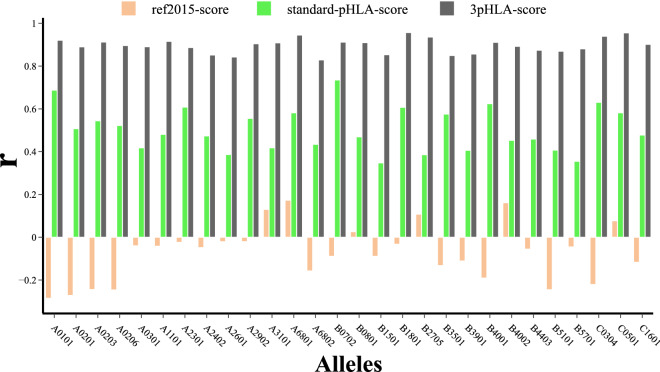
The predictive power of ref2015-score, standard-pHLA-score, and 3pHLA-score is evaluated and compared on the test portion of Dataset 1. Results are reported for individual alleles, listed on the x-axis. The regression power of the scores is quantified using Pearson’s *r*, on the y-axis.

First, we compare the regression and classification power of the following scoring functions on the test portion of Dataset 1: ref2015-score, standard-pHLA-score, and 3pHLA-score. We are interested to see how well the rank of predicted binding affinities matches the rank of the true binding affinity values for tested pHLA complexes. On the other hand, with the classification metrics (AUROC, AUPRC), we want to test how well predicted binding affinities separate the known binders from non-binders. The regression power of the scoring functions is evaluated on the test set using Pearson’s correlation coefficient *r* (Fig. 2). 3pHLA-score outperformed both ref-2015 and standard-pHLA-score: while 3pHLA-score achieves an average Pearson’s correlation of 0.75 on the test set, ref2015-score and standard-pHLA-score achieve a significantly lower correlation of 0.09 and 0.46, respectively (Table 1). Figure S3 shows in detail the correlation between predicted and experimental scores for the best and worst performing 3pHLA-score models. The same pattern is observed for all individual HLA alleles across all investigated metrics (Fig. S2, Table S2). Additionally, we provide the same analysis for standard-pHLA-score and 3pHLA-score that are trained using alternative ML regression techniques (Supplementary Material subsection *Alternative ML regression techniques*). 3pHLA-score consistently outperforms standard-pHLA-score across all ML regression techniques we assessed.

**Table 1:** Results of scoring functions obtained using different training protocols on the test set averaged across all HLA alleles for all four evaluated metrics (Pearson’s correlation coefficient |*r*|, Spearman’s correlation coefficient $|\rho |$, the Area Under the Receiver Operator Curve AUROC and the Area Under the Precision Recall Curve AUPRC) . The highest values and best performing values in each column are bolded.

	|*r*|	$|\rho |$	AUROC	AUPRC
3pHLA-score	**0.75**	**0.90**	**0.98**	**0.99**
standard-pHLA-score	0.46	0.50	0.80	0.82
ref2015-score	0.09	0.07	0.44	0.56

### The predictive power of the per-peptide-position protocol varies depending on the choice of positions

**Figure 3:**
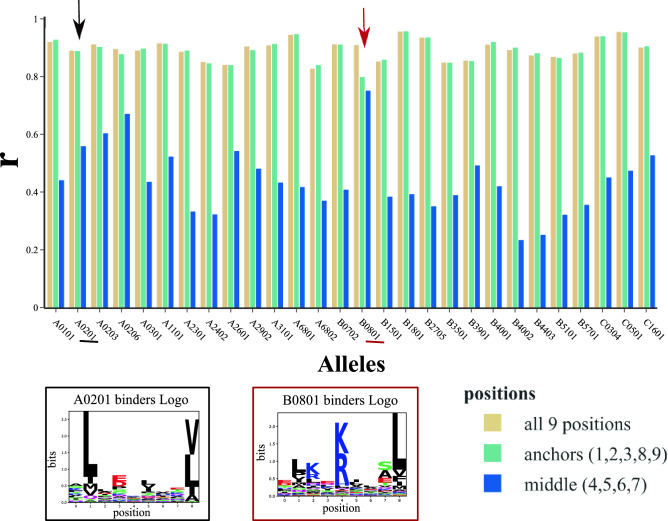
Results of the ablation study, in which 3pHLA-score was trained using different subsets of peptide positions: all nine positions, anchor positions (1, 2, 3, 8, 9), and middle positions (4, 5, 6, 7). Results are reported for individual alleles indicated on the x-axis. The regression power of scoring functions is quantified using Pearson’s *r* and plotted on the y-axis. The logo representation of the HLA-A0201 and HLA-B0801 binders is presented to compare the importance of the middle position 5 for HLA-B0801.

We know that different residue positions in a peptide (i.e., peptide positions) have different contributions to HLA binding and T-cell recognition. While middle positions are usually more exposed and therefore involved in the recognition by T-cells, the anchor positions are usually buried in the HLA groove and play a more direct role in pHLA binding^55^. For this reason, we conducted an ablation study to investigate the influence of different peptide positions on the performance of 3pHLA-score. 3pHLA-score was trained with three different position sets: all nine positions, anchor positions (1, 2, 3, 8, 9), or middle positions (4, 5, 6, 7). We generate binding affinity predictions for the test set using these different versions of 3pHLA-score and we investigate how well the affinities are ranked compared to the true affinities as well as how well the predictions separate binders from non-binders. We observe that the choice of positions in 3pHLA-score has a substantial influence on its performance on the test set according to Pearson’s correlation (Fig. 3 , Table S3) and other metrics (Figure S4). The performance of training with anchor positions only and all nine positions is comparable, with *r* values higher than 0.8 for most HLA alleles. The *r* values drop below 0.7 when middle positions only are used. The only exception is the HLA-B0801 allele, for which closer inspection of the binding motif in IEDB (iedb.org/mhc/252) clearly indicates the importance of position 5 for peptide binding, as reflected in the HLA-B0801 predictor’s performance.

### 3pHLA-score outperforms well-validated structure-based scoring functions in an epitope discovery setting

The goal of structure-based virtual screening for epitope discovery is to distinguish true binder peptides from non-binders, which can be seen as a classification problem. To evaluate 3pHLA-score in an epitope discovery scenario, we compare it to a variety of widely used structural scoring functions (Table S5) on a dataset containing 100 strong binders and 2,000 decoys across 16 HLA alleles (Dataset 2). Our results show that 3pHLA-score clearly outperforms other evaluated scoring functions in this virtual screening setting, with an average AUPRC of 0.71 compared to the second-best scoring function (Vinardo) with AUPRC of 0.35 (Table 2). This is consistent with 3pHLA-score achieving higher values of both AUROC and AUPRC for all investigated HLA alleles individually (Tables S7, S8), and 3pHLA-score separating binders from non-binders more clearly than other scoring functions (Figure S5). It is also important to note that Dataset 2 was not used in the training phase. Therefore, this experiment also demonstrates the capacity of 3pHLA-score to generalize to new datasets.

**Table 2:** AUROC and AUPRC values aggregated for the virtual screening experiment across HLA alleles. The highest values and best performing values in each column are bolded.

	AUROC	AUPRC
3pHLA-score	**0.977**	**0.712**
Vinardo^40^	0.898	0.354
Vina^39^	0.871	0.291
GradDock^31^	0.778	0.182
DOPE^41^	0.769	0.141
AutoDock4^38^	0.751	0.141
FoldX^42^	0.687	0.142

In the context of epitope discovery, current pipelines use sequence-based scoring functions. Therefore, we evaluate how 3pHLA-score compares to sequence-based methods and present the details of this analysis in Supplementary Material. Overall, 3pHLA-score has comparable performance to selected sequence-based methods with an average AUROC of 0.977 compared to the best achieved AUROC of 0.993 with MHCFlurry2.0^10^. Note that we do not know if MHCFlurry2.0 has had a part of our test dataset in their training, which might give it a slight advantage.

### 3pHLA-score can generalize to an independent dataset

We tested the ability of 3pHLA-score to generalize to other “types“ of structural data with the independent Dataset 3. Dataset 1 and Dataset 2 contain structures that were all modeled by APE-Gen^28^ with peptides of 9 residues in length (9-mers). With an independent dataset, we can investigate the possible biases towards this modeling tool and explore how to generalize to the peptides of length 10. Dataset 3 contains experimentally resolved three-dimensional pHLA structures involving binders and non-binders modeled by Docktope^54^. Importantly, it contains 10-mer peptides. As 3pHLA-score was trained on 9-mers, the size of the input of the model is $9 \times 19$ (i.e., 9 peptide positions times 19 energy terms). To score 10-mers, we excluded the energy terms of the middle position (i.e., position 6) of the peptide. The rationale for this approach lies in the aforementioned experimental findings on peptide anchors^46^.

Since Dataset 3 contains peptides with a wide range of experimental binding affinities (strong, medium, weak binders, and non-binders), two tasks were identified for the scoring functions: a regression and a classification task. For the regression task, scoring functions are expected to predict the correct peptide ranking in terms of binding affinities. In this context, it is also interesting to analyze the range of scores predicted for a given peptide within different structures (i.e., same complex, but different crystallography experiments). The smaller the range, the more consistent a scoring function is for scoring a certain peptide. For the classification task, we label peptides with three different binding affinity thresholds: 50 nM (distinguishing strong binders from others), 500 nM (distinguishing strong and medium binders from others), and 25,000 nM (distinguishing binders from non-binders). The classification power of scoring functions was evaluated using AUROC and AUPRC.

**Figure 4:**
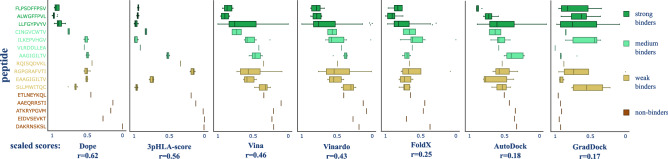
Performance of different scoring functions in evaluating the binding affinity of structures from the independent Dataset 3. Pearson’s correlation coefficient is indicated next to the name of the scoring function. Peptides involved in the structures of Dataset 3 (see Table S4) are listed on the y-axis. The peptide names and corresponding box plots are colored and arranged along the y-axis according to their experimental binding affinity (ranging from dark green, strong binders, at the top, to dark orange, non-binders, at the bottom). Predicted scores scaled to the range 1-0 are plotted on the x-axis (1-highest predicted binder; 0-non-binder). The correlation is calculated for the predicted binding affinity of each of the 82 structures present in Dataset 3 with respect to their experimental binding affinities.

The scaled scores aggregated across structures for each peptide are shown in Fig. 4. The scaled score for each structure in Dataset 3 is shown in Figure S6. Pearson’s correlation coefficient between experimental binding affinity and predicted scores is given in Table S6. While DOPE scoring function consistently outperforms others, 3pHLA-score shows competitive performance in this challenging setting and is a runner-up in most of the evaluated tasks. In the regression setting, this fact is reflected by DOPE achieving a correlation of 0.62, while 3pHLA-score achieves a correlation of 0.56 with experimental affinity. However, neither of these correlations are strong. On the other hand, both DOPE and 3pHLA-score produce small variations of the score for different structures of the same peptide which is a desirable property for an epitope discovery task. With respect to the classification task, DOPE produced the best results according to AUROC and AUPRC for most of the thresholds analyzed (Table 3). The 3pHLA-score also occupied a position of relevance, having the best AUPRC value for the 500 nM threshold and the second-best AUPRC values for the 50 nM and 25,000 nM thresholds. When considering AUROC values, Vina and Vinardo are the second best for the 50 nM and 500 nM thresholds; the 3pHLA-score was again the second best for the 25,000 nM threshold.

**Table 3:** Quantified power of scoring functions to discriminate between peptides of different binding strength on the Dataset 3.

	thr = 50 nM		thr = 500 nM		thr = 25,000 nM	
	AUROC	AUPRC	AUROC	AUPRC	AUROC	AUPRC
DOPE	**1.0**	**1.0**	**0.84**	**0.69**	**1.0**	**0.97**
3pHLA-score	0.76	**0.85**	0.76	**0.72**	**0.99**	** 0.91**
Vina	0.84	0.71	**0.81**	0.52	0.90	0.27
Vinardo	**0.85**	0.73	0.78	0.49	0.87	0.23
FoldX	0.83	0.74	0.70	0.41	0.83	0.19
AutoDock4	0.73	0.63	0.63	0.35	0.82	0.17
GradDock	0.42	0.48	0.48	0.33	0.16	0.04

$^{*}$ Top 2 performing values are bolded.

$$^{**}$$ thr: threshold of binding affinity used to label different classes.

## Discussion

Motivated by experimental findings of peptide anchors, we hypothesize that important information for training ML pHLA scoring functions is lost in standard training protocols. We try to recover this information using our novel per-peptide-position protocol and we apply it to develop the 3pHLA-score.

In the first set of experiments, we show how energy decoupling of the per-peptide-position protocol (as applied to 3pHLA-score) significantly increases the predictive power of models (Figs. 2,  S2, Table 1). Furthermore, we show that the predictive power of 3pHLA-score is highly dependent on the choice of peptide positions to be decoupled (Figs. 3, S4).

Next, we provide an extensive comparison of the 3pHLA-score against other widely used scoring functions. 3pHLA-score shows a clear superior performance to other scoring functions when tested in the epitope discovery setting where we perform structure-based virtual screening of true peptide-binders to HLA receptors (Table 2, Fig. S5).

Note that the training of the 3pHLA-score could not have been done using only experimentally-determined crystal structures, due to the limited number of pHLA crystals available (i.e., less than 800 in the PDB). Therefore, we chose to use models produced by APE-Gen, which is potentially the only currently available pHLA-specific modeling tool with the capacity to model thousands of complexes (e.g., nearly 80,000 complexes modeled for Dataset 1). The choice of the modeling method, however, can introduce a bias in the training of the scoring function. To test that, we used an independent dataset (i.e., Dataset 3) containing crystal structures and models produced by a different tool DockTope. Note that DockTope uses a very different modeling protocol, based on fixed backbone templates. Despite involving different types of structures, our results still show a good overall performance of 3pHLA-score on Dataset 3, being competitive with other popular scoring functions. These results suggest that 3pHLA-score can be used with crystal structures and models produced by other tools, without additional training, although a broader survey with other tools for pHLA modeling and peptide-docking will be needed to further corroborate this point. Interestingly, in this experiment, the most consistent predictions across different structures of the same complex, and the strongest correlation with experimental data, were observed for DOPE (Table 3, Fig. 4). This surprising result might be directly linked to the nature of this dataset and the intended use of DOPE. DOPE scoring function is a statistical potential used to assess the global quality of homology models produced by Modeller^56^. This provides two advantages to DOPE in the experiment with Dataset 3. First, this dataset is mostly composed of crystal structures, and DOPE’s global assessment was observed in our experiment to be more resilient to small differences between different conformations of the same complex. Second, DOPE is well suited to distinguish the non-binders, which were modeled with a docking-based approach, from the experimentally-determined crystal structures used for all other complexes. Our results show that the 3pHLA-score predictions could be generalized to both DockTope models and crystal structures, while the good performance of DOPE did not generalize to other datasets. For instance, 3pHLA-score outperformed DOPE and other scoring functions on Dataset 2 (Table 2, Fig. S5). It is therefore the method that provides the most consistent results across the three different datasets.

The discovered potential of per-peptide-position energy terms for pHLA system opens up many additional opportunities that we discuss here. To build 3pHLA-score we trained separate models for each HLA allele. This limits the use of 3pHLA-score to a fixed set of HLA alleles that is found in the training dataset. However, a bigger pan-allele dataset can be acquired in the future and the same method could be applied to train a more general pan-allele model. APE-Gen, the tool used here to model pHLA structures, is currently limited to modeling the peptides containing only the 20 standard amino acids. Therefore, modeling phosphorylated peptides (or peptides with other post-translational modifications) and assessing the HLA-binding energies of these peptides with 3pHLA-score is another interesting challenge, which would greatly broaden the impact of our methods on ongoing efforts in epitope discovery^57^. 3pHLA-score was trained here with a single conformation per peptide, to predict HLA binding affinity in the context of structural virtual screening. Future studies could investigate the use and refinement of 3pHLA-score to the geometry prediction task (i.e., ranking different conformations of the same pHLA complex). For that task, we would propose using the same per-peptide-position training protocol on a dataset that contains multiple conformations per peptide mapped to a corresponding experimentally determined crystal structures. The baseline scoring function for extracting the energy terms used here was ref2015. Therefore, it remains to be determined how the same training protocol would perform when applied to another existing scoring function which provides energy terms for specific regions of the model. This question is left for future work. As discussed above, our per-peptide-position protocol could provide more opportunities than exemplified by 3pHLA-score. The protocol can be applied beyond the ref2015 energy terms as well as beyond the pHLA system. For that reason, we make a distinction between the 3pHLA-score and the per-peptide-position protocol.

Overall, our results confirm that important structural signal for binding prediction gets lost when the standard energy terms are calculated at the all-peptide-atom level. This could point to the fact that the additive nature of the standard all-atom energy terms is not appropriate for the pHLA system. Our work emphasizes how experimental findings can help engineer more powerful features and train ML models with better predictive power. This can serve as a guideline for future attempts of training custom ML scoring functions for different systems of interest. As more structural pHLA data become available, we hope that our findings will inspire future efforts in training structure-based pHLA binding predictors that could enter epitope discovery pipelines and complement sequence-based methods. 3pHLA-score has direct application to epitope discovery projects, which could help advance the development of vaccines against several types of cancer and viral infections.

## Supplementary Information

Supplementary Information.

## Data Availability

Sequencing data was not generated in this study. The code and the data used for running the experiments and training along with the scoring function and datasets is available in the repository: https://github.com/KavrakiLab/3pHLA-score.
