# Supplementary material for: 3pHLA-score improves structure-based peptide-HLA binding affinity prediction
Source: Sci Rep. 2022 Jun 24;12:10749. doi: 10.1038/s41598-022-14526-x (PMC9232595; doi:10.1038/s41598-022-14526-x)
Supplement: Supplementary file 1 — Supplementary Information. [file 41598_2022_14526_MOESM1_ESM.pdf]

3pHLA-score improves structure-based peptide-HLA binding affinity prediction

Anja Conev, Didier Devaurs, Mauricio Menagatti Rigo, Dinler Amaral Antunes, Lydia Kavraki

Supplementary material

Peptide-HLA structure terminology

The HLA class I receptor is a heterodimer composed of a heavy chain (α) and a light chain ($beta_2$-microglobulin) (depicted in Figure S1a). The light chain is not highly variable and is not encoded by the HLA gene. The heavy chain is highly variable and contains three regions (α1, α2, α3). Regions a1 and a2 form the binding site. The binding site is the groove between a1 and a2 helices where peptides bind to HLAs and form the peptide-HLA complex (Figure S1b). Within the binding site of class I HLA receptors, six smaller pockets are defined. These pockets are labeled A to F, with pockets B and F accommodating the so-called anchor positions at the N-terminus and C-terminus of the peptide, respectively[55] (Figure S1c). Peptides that bind to HLA class I molecules are usually 9-11 amino acids long. pHLA binding is conditioned by the structural and energy fit of peptides to the binding site.

Evaluation metrics

To assess the regression power of scoring functions, the following metrics are used:

- Pearson's correlation coefficient r

$$r = \frac{cov(Y,Y')}{\sigma_Y \sigma_{Y'}} \tag{S.1}$$

where Y are the observed values and Y' the models' predictions, cov is covariance and σ is the standard deviation. r quantifies the linear relationship between the observed and predicted values. The observed values in our case are the experimental binding affinities of the pHLA complex that are considered as labels in the dataset. The predicted values are values given by different scores that we evaluate (3pHLA-score, standard-pHLA-score, Vina, Vinardo). r ranges from -1 to 1 and the relationship is considered to be strong when the absolute value $|r|$ is above 0.7.

- Spearman's correlation coefficient ρ

$$\rho = \frac{cov(rk_Y, rk_{Y'})}{\sigma_{rk_Y} \sigma_{rk_{Y'}}} \tag{S.2}$$

where rk_Y are the ranks of the observed values and $rk_{Y'}$ the ranks of predictions, cov is covariance and σ is the standard deviation. ρ quantifies the monotonic relationship between the observed and predicted values. The observed values in our case are the experimental binding affinities of the pHLA complex that are considered as labels in the dataset. The predicted values are values given by different scores that we evaluate (3pHLA-score, standard-pHLA-score, Vina, Vinardo). ρ ranges from -1 to 1 and the relationship is considered to be strong when the absolute value $|\rho|$ is above 0.7.

Note that different scoring functions output binding affinities in different units (i.e., GradDock predicts binding affinity in nM, AutoDock4 output is in kcal/mol). Not all units were consistent with our labels (i.e., nM). Additionally, the primary use of the scoring functions in virtual screening tasks is to correctly rank the scored structures. This is why we use correlation metrics rather than coefficient of determination to compare regression power of the scoring functions.

To assess the power of scoring functions to make a distinction between the binders and non-binders (classification power) the following metrics are used:

- Area Under the Receiver Operating Characteristic (AUROC) which is the area under the curve when the true positive rate is plotted against the false positive rate with varying thresholds. It gives an estimate of how well models can rank the examples. To calculate AUROC we use the binary labels for binding and non-binding pHLA complex and the predicted scores (3pHLA-score, standard-pHLA-score, Vina, Vinardo). AUROC ranges from 0 to 1, and values closer to 1 correspond to better predictive power.

- Area Under the Precision-Recall Curve (AUPRC) which is the area under the curve when the precision is plotted against recall with varying thresholds. It gives an estimate of whether models can correctly identify the positive examples without predicting too many false positives. Unlike AUROC, AUPRC is robust to imbalanced datasets. To calculate AUPRC we use the binary labels for binding and non-binding pHLA complex and the predicted scores (3pHLA-score, standard-pHLA-score, Vina, Vinardo). AUPRC ranges from 0 to 1, and values closer to 1 correspond to better predictive power.

Label transformation

$$x_{transformed} = \begin{cases} 1 - log_{50000}(x), & \text{when } x \leq 50000 \\ 0, & \text{otherwise} \end{cases} \tag{S.3}$$

where x is the binding affinity label expressed in nM units.

Per-peptide-position feature vector

To extract the per-peptide-position features we follow a protocol described in the subsection *3pHLA-score* of the section *Methods*. The final per-peptide-position feature vector has the following form:

$$\begin{bmatrix} fa\_atr_1 & fa\_rep_1 & fa\_intra\_rep_1 & ... & ref_1 \\ fa\_atr_2 & fa\_rep_2 & fa\_intra\_rep_2 & ... & ref_2 \\ \vdots & & & \vdots & \\ fa\_atr_N & fa\_rep_N & fa\_intra\_rep_N & ... & ref_N \end{bmatrix}_{N\times 19} \tag{S.4}$$

Each column corresponds to a ref2015 energy term (listed in Table S1). Each row corresponds to a different peptide residue position (as indicated by the numerical index). For example - the first row contains all the energy terms (fa_atr, fa_rep, fa_intra_rep, ..., ref) for the residue at position 1 of the peptide. N is the number of peptide residues.

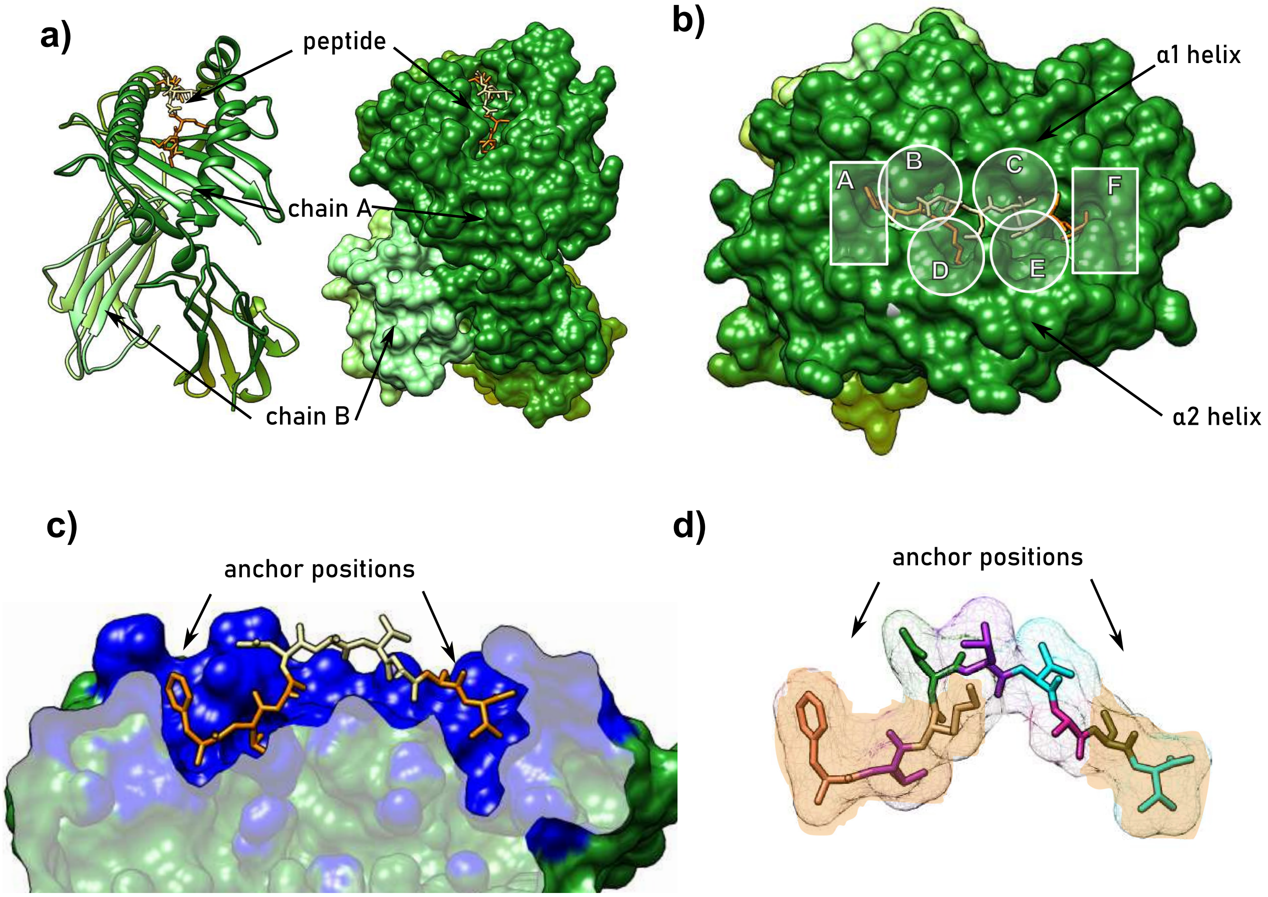

Figure S1. Basic terminology of the pHLA complex introduced using the HLA-A0201 receptor with the FLKDLVASV peptide bound. **(a)** Ribbon (left) and surface (right) representations of the complex (HLA receptor in green and peptide in orange); both chains A (dark green) and B (light green) are indicated. The anchor positions of the peptide are highlighted with a darker orange shade. **(b)** Zoomed-in view of the binding site with $\alpha 1$ and $\alpha 2$ helices indicated as well as 6 known pockets of the binding site (A-F). **(c)** A cross-section of the binding site (colored in blue) shows the depth of pockets A and F, as well as anchor positions within the peptide (orange). **(d)** Peptide representation without the HLA binding site each position within the peptide is colored with a different color. Surface around the peptide is represented with a colored mash. The anchor positions (positions 1, 2, 3, 8, 9) of the peptide are indicated with the orange shade.

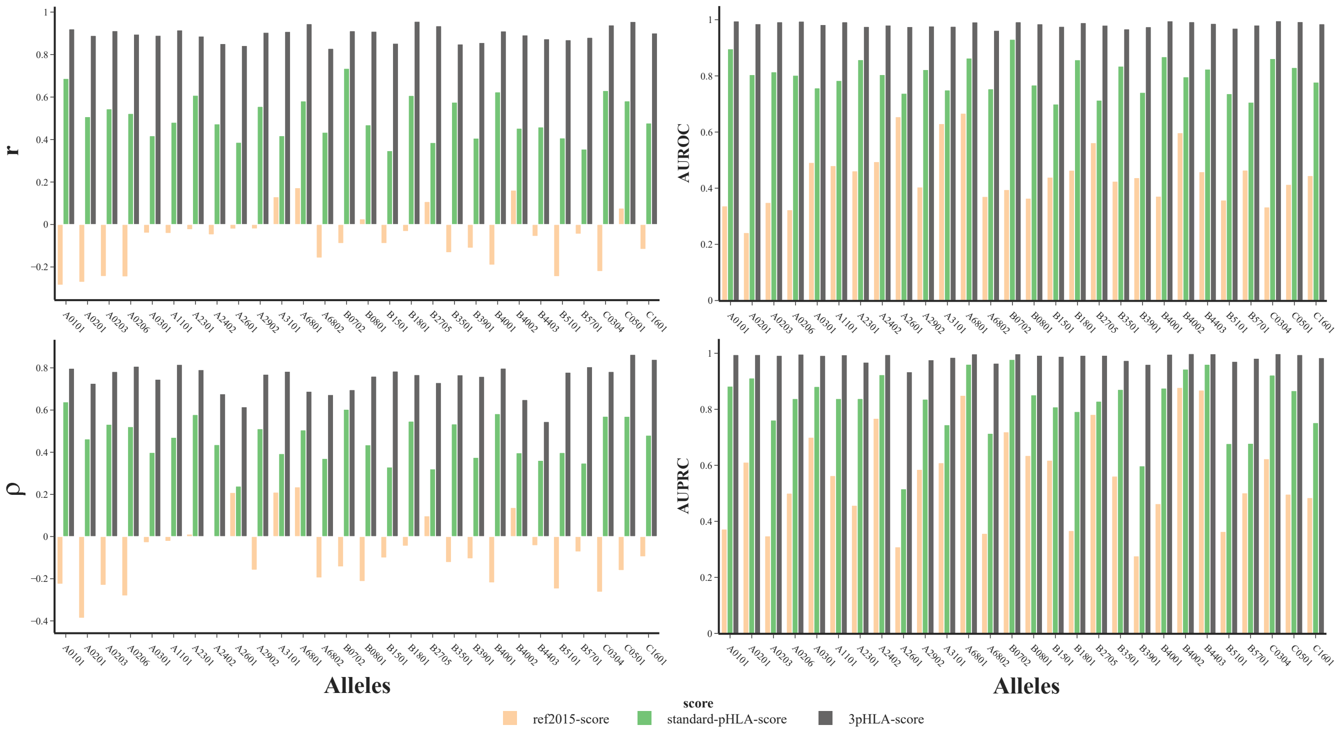

Figure S2. Three scoring functions (ref2015-score, standard-pHLA-score and 3pHLA-score) are evaluated and compared in a per-allele setting on the test portion of Dataset 1 - alleles are indicated on the x-axis. The regression power of the scoring functions is quantified with Pearson's r and Spearman's ρ metrics and plotted on the y-axis. The classification power of the scoring functions is quantified with the $AUROC$ and $AUPRC$ metrics and plotted on the y-axis. 3pHLA-score clearly outperforms ref2015-score and standard-pHLA-score.

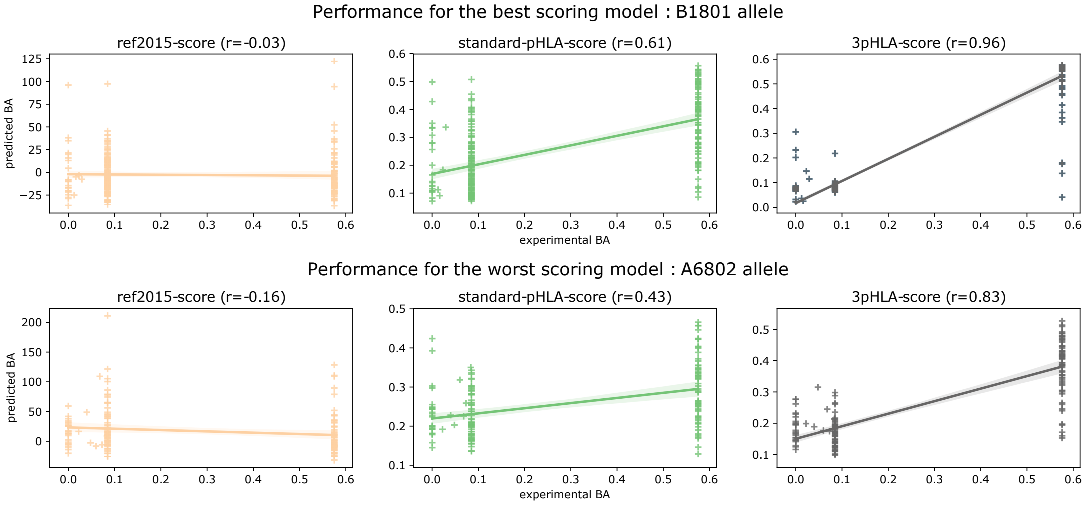

Figure S3. Scatter plot showing the correlation between experimental and predicted binding affinities for the three scoring functions (ref2015-score, standard-pHLA-score and 3pHLA-score). The predictions are made on the test portion of Dataset 1. The composition of the test set is such that the experimental binding affinities of binders are skewed around 0.6 while the values of non-binders range from 0.0 to 0.1. Here we present the results for two representative alleles - one for which the 3pHLA-score performs best (B1801 allele) and one where 3pHLA-score has the worst performance of all trained models (A6802 allele).

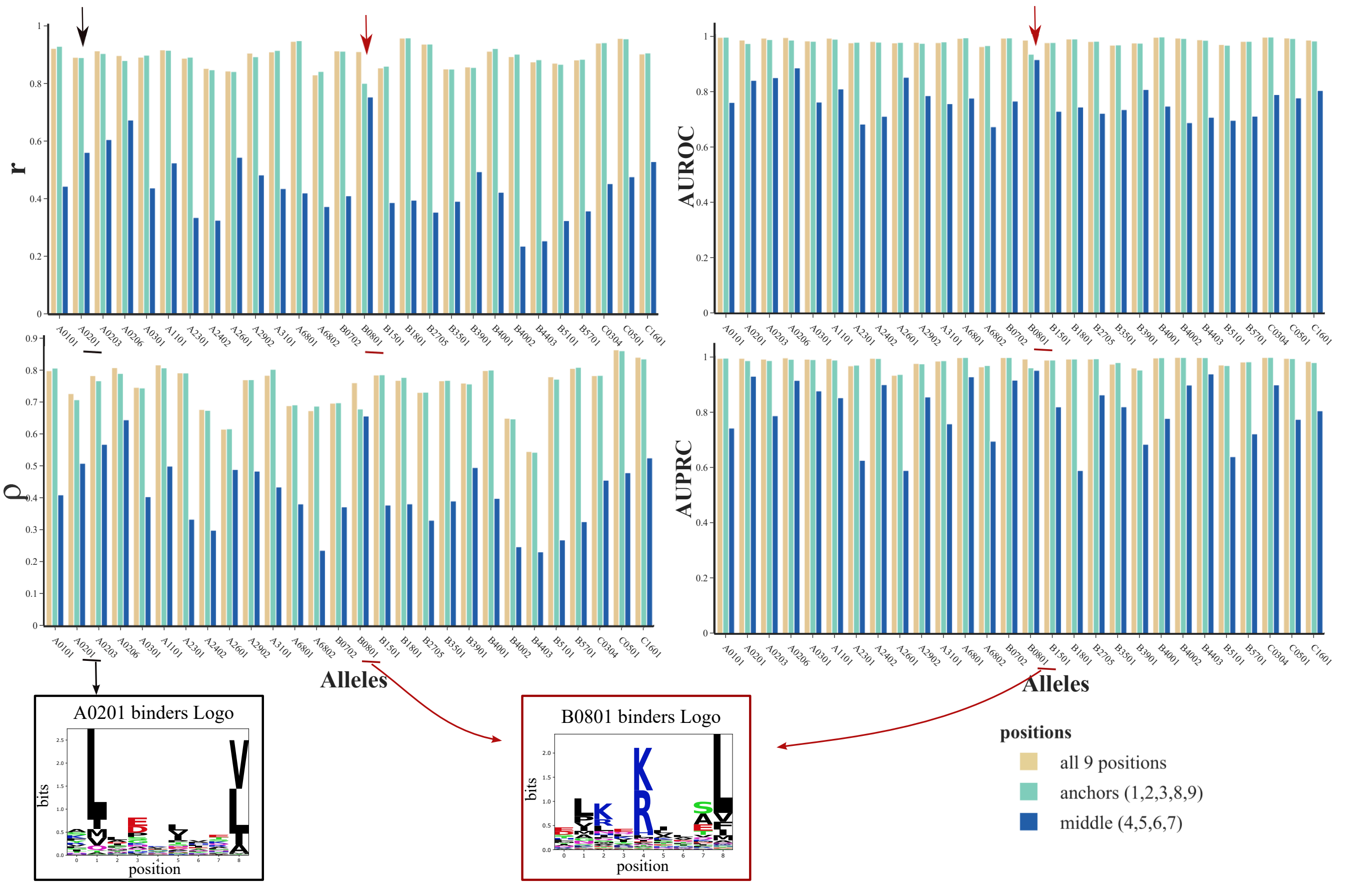

Figure S4. 3pHLA-scores trained with different peptide positions are compared (all 9 positions, anchor positions or middle positions) in a per-allele setting - alleles are indicated on the x-axis. The regression power of the scoring functions is quantified with Pearson's r and Spearman's ρ metrics and plotted on the y-axis. The classification power of the scores is quantified with $AUROC$ and $AUPRC$ and plotted on the y-axis. The predictive power of 3pHLA-score is similar when anchor positions are used instead of all 9 positions, while it drops when using only middle positions. The only exception is the HLA-B0801 allele: it is known to have a less common binding pattern, with a dominant anchor at position 5, which is depicted in the logo representation of its peptide binders compared to one of the more usual motifs, such as HLA-A0201.

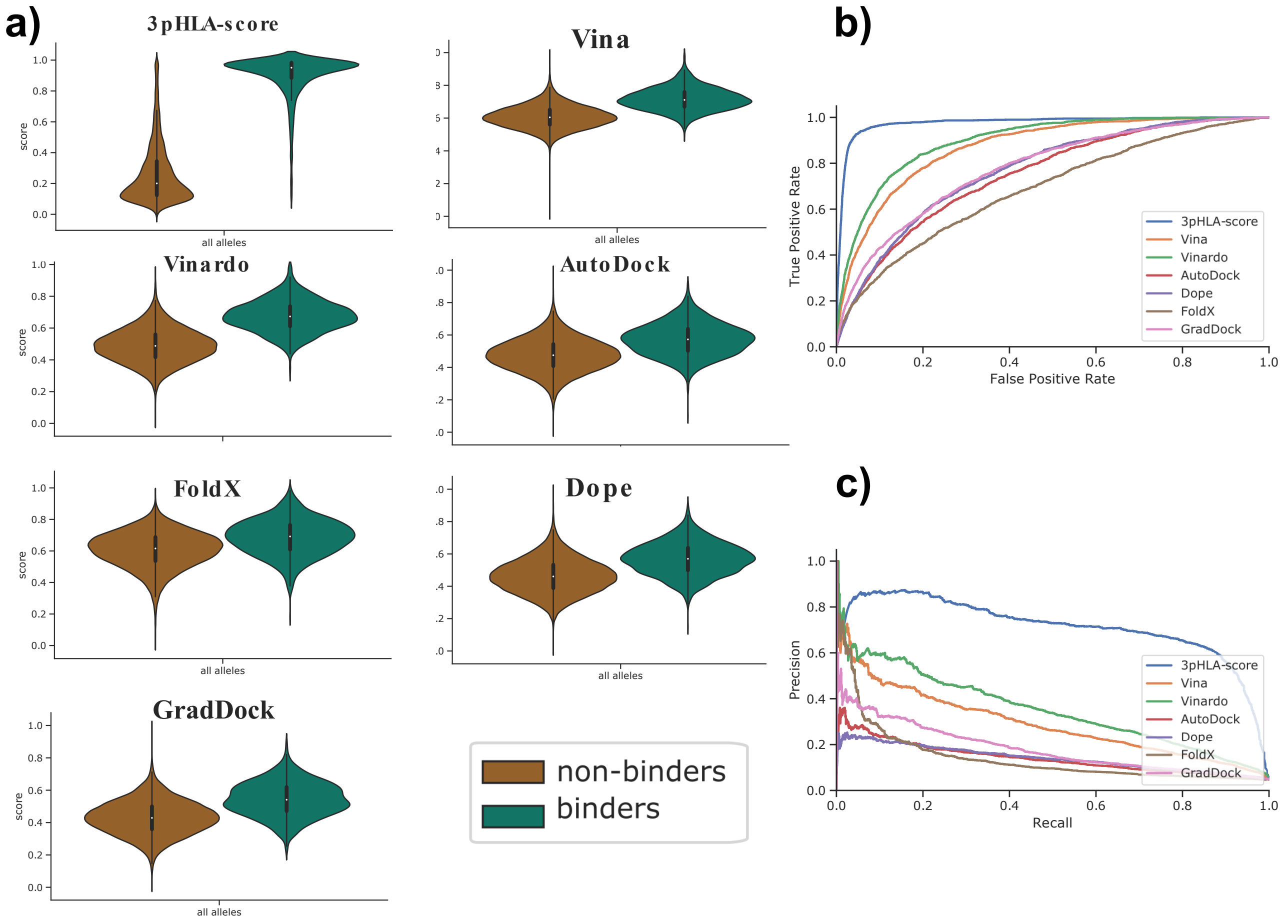

Figure S5. Performance of different scoring functions (listed in Table S5) in the virtual screening setting. Results are aggregated across alleles. a) Violin plots show the distribution of predicted binding affinities for binders (green) and non-binders(brown) and give an estimate of how well different scoring functions distinguish binders from non-binders in this setting. b) ROC-curves for different scoring functions in the virtual screening setting; c) PR-curves for different scoring functions in the virtual screening setting.

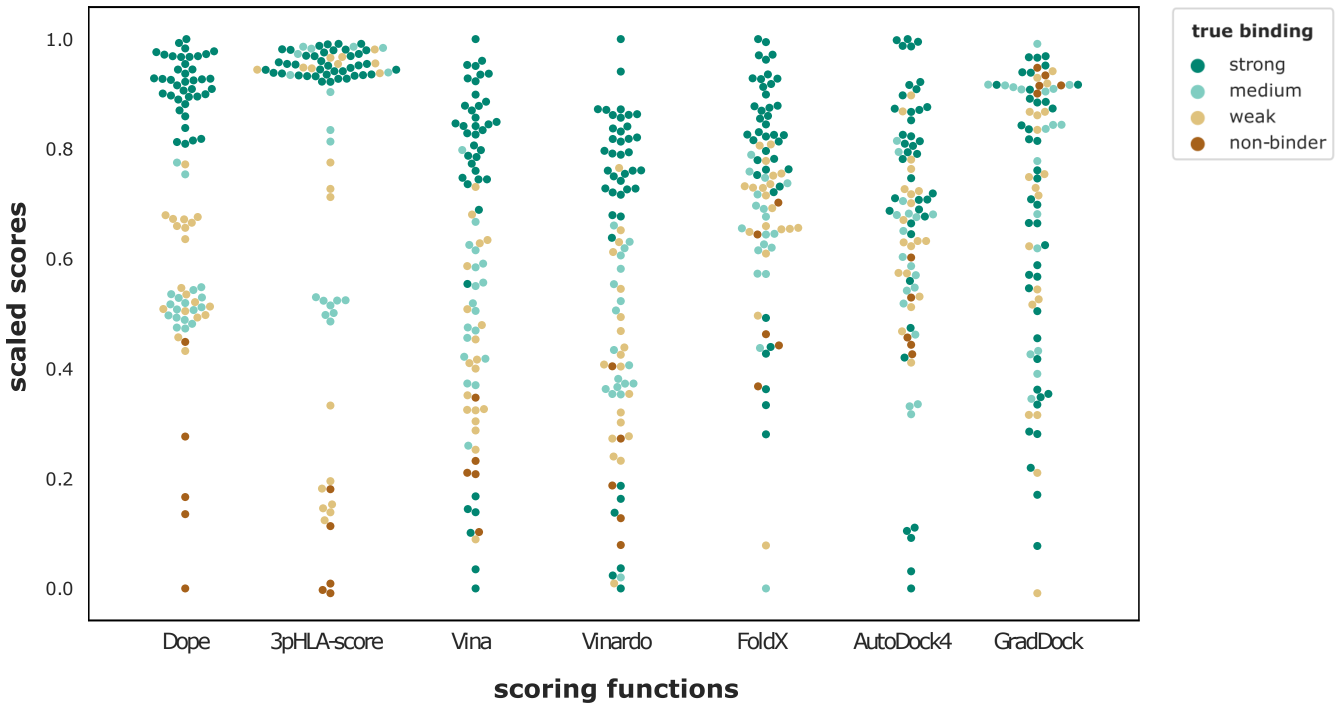

Figure S6. Scaled scores given by different scoring functions to structures from Dataset 3. The scores are scaled to fit 0-1 range and plotted on the y-axis. Each point represents a single structure and is colored based on the strength of experimental binding affinity.

Supplementary Table S1. Rosetta ref2015 energy terms[37]

Term	Description
fa_atr	Attractive energy between two atoms on different residues separated by distance, d
fa_rep	Repulsive energy between two atoms on different residues separated by distance, d
fa_intra_rep	Repulsive energy between two atoms on the same residue, separated by distance, d
fa_sol	Gaussian exclusion implicit solvation energy between protein atoms in different residues
lk_ball_wtd	Orientation-dependent solvation of polar atoms assuming ideal water geometry
fa_intra_sol	Gaussian exclusion implicit solvation energy between protein atoms in the same residue
fa_elec	Energy of interaction between two non-bonded charged atoms separated by distance, d
hbond_lr_bb	Energy of short range hydrogen bonds
hbond_sr_bb	Energy of long range hydrogen bonds
hbond_bb_sc	Energy of backbone-side chain hydrogen bonds
hbond_sc	Energy of side chain to side chain hydrogen bonds
dslf_fa13	Energy of disulfide bridges
rama_prepro	Probability of backbone ϕ,ψ angles given amino acid type
p_aa_pp	Probability of amino acid identity given backbone ϕ,ψ angles
fa_dun	Probability that a chosen rotamer is native-like given backbone ϕ,ψ angles
omega	Backbone-dependent penalty for cis ω dihedrals that deviate from 0 and trans ω dihedrals that deviate from 180
pro_close	Penalty for an open proline ring and proline ω bonding energy
yhh_planarity	Sinusoidal penalty for non-planar tyrosine X^3 dihedral angle
ref	Reference energies for amino acid types

Supplementary Table S2. Pearson's correlation coefficient and corresponding two-sided p-values between the experimental binding affinities and predicted binding affinities. The results are evaluated on the test portion of Dataset 1, as part of the set of experiments where we compare different training protocols and are reported for each allele.

	ref2015-score		standard-pHLA-score		3pHLA-score	
Allele	r	p-value	r	p-value	r	p-value
A0101	-0.29	$4.36*10^{-7}$	0.69	$1.07*10^{-43}$	0.92	$4.18*10^{-125}$
A0201	-0.27	$9.15*10^{-15}$	0.51	$1.33*10^{-52}$	0.89	$3.85*10^{-269}$
A0203	-0.24	$3.86*10^{-5}$	0.54	$9.01*10^{-23}$	0.91	$1.37*10^{-108}$
A0206	-0.25	$3.48*10^{-3}$	0.52	$4.15*10^{-11}$	0.90	$3.90*10^{-50}$
A0301	-0.04	$3.38*10^{-1}$	0.42	$1.95*10^{-25}$	0.89	$5.25*10^{-196}$
A1101	-0.04	$4.87*10^{-1}$	0.48	$1.65*10^{-17}$	0.92	$2.68*10^{-111}$
A2301	-0.02	$7.47*10^{-1}$	0.61	$6.80*10^{-20}$	0.89	$1.36*10^{-62}$
A2402	-0.05	$4.25*10^{-1}$	0.47	$9.13*10^{-17}$	0.85	$1.59*10^{-78}$
A2601	-0.02	$7.47*10^{-1}$	0.39	$9.87*10^{-10}$	0.84	$5.92*10^{-64}$
A2902	-0.02	$7.76*10^{-1}$	0.56	$4.07*10^{-17}$	0.90	$4.98*10^{-73}$
A3101	0.13	$7.80*10^{-2}$	0.42	$3.59*10^{-9}$	0.91	$5.62*10^{-71}$
A6801	0.17	$7.65*10^{-3}$	0.58	$7.65*10^{-23}$	0.95	$2.92*10^{-116}$
A6802	-0.16	$4.38*10^{-2}$	0.43	$6.39*10^{-09}$	0.83	$9.97*10^{-43}$
B0702	-0.09	$4.90*10^{-2}$	0.73	$1.16*10^{-83}$	0.91	$1.73*10^{-189}$
B0801	0.03	$6.59*10^{-1}$	0.47	$3.26*10^{-17}$	0.91	$1.66*10^{-111}$
B1501	-0.09	$1.13*10^{-1}$	0.35	$2.19*10^{-10}$	0.85	$1.02*10^{-90}$
B1801	-0.03	$5.99*10^{-1}$	0.61	$8.40*10^{-28}$	0.96	$4.43*10^{-141}$
B2705	0.11	$4.82*10^{-2}$	0.39	$2.32*10^{-13}$	0.94	$5.68*10^{-153}$
B3501	-0.13	$3.28*10^{-2}$	0.58	$2.35*10^{-24}$	0.85	$1.19*10^{-73}$
B3901	-0.11	$1.65*10^{-1}$	0.41	$1.17*10^{-7}$	0.86	$1.17*10^{-46}$
B4001	-0.19	$7.82*10^{-3}$	0.62	$3.12*10^{-22}$	0.91	$2.19*10^{-75}$
B4002	0.16	$7.80*10^{-2}$	0.45	$2.04*10^{-7}$	0.89	$1.53*10^{-42}$
B4403	-0.06	$3.63*10^{-1}$	0.46	$2.00*10^{-15}$	0.87	$1.04*10^{-85}$
B5101	-0.25	$5.66*10^{-5}$	0.41	$5.67*10^{-12}$	0.87	$2.88*10^{-82}$
B5701	-0.05	$3.95*10^{-1}$	0.35	$4.61*10^{-12}$	0.88	$1.18*10^{-117}$
C0304	-0.22	$1.01*10^{-3}$	0.63	$1.45*10^{-25}$	0.94	$2.93*10^{-102}$
C0501	0.08	$2.52*10^{-1}$	0.58	$1.36*10^{-21}$	0.96	$4.14*10^{-119}$
C1601	-0.12	$9.10*10^{-2}$	0.48	$2.31*10^{-13}$	0.90	$1.39*10^{-77}$

Supplementary Table S3. Pearson's correlation coefficient and corresponding two-sided p-values between the experimental binding affinities and predicted binding affinities. The results are evaluated on the test portion of Dataset 1, as part of the set of experiments where we compare 3pHLA-score trained on different sets of residue positions.

	all 9 positions		anchor positions (1,2,3,8,9)		middle positions (4,5,6,7)	
Allele	r	p-value	r	p-value	r	p-value
A0101	0.92	$4.18*10^{-125}$	0.93	$4.01*10^{-131}$	0.44	$5.49*10^{-16}$
A0201	0.89	$3.85*10^{-269}$	0.89	$8.87*10^{-268}$	0.56	$4.37*10^{-66}$
A0203	0.91	$1.37*10^{-108}$	0.90	$5.66*10^{-103}$	0.60	$5.24*10^{-29}$
A0206	0.90	$3.90*10^{-50}$	0.88	$8.03*10^{-46}$	0.67	$1.26*10^{-19}$
A0301	0.89	$5.25*10^{-196}$	0.90	$1.91*10^{-203}$	0.44	$6.01*10^{-28}$
A1101	0.92	$2.68*10^{-111}$	0.91	$2.63*10^{-110}$	0.52	$5.39*10^{-21}$
A2301	0.89	$1.36*10^{-62}$	0.89	$9.35*10^{-64}$	0.33	$3.66*10^{-6}$
A2402	0.85	$1.59*10^{-78}$	0.85	$8.94*10^{-77}$	0.33	$3.47*10^{-8}$
A2601	0.84	$5.92*10^{-64}$	0.84	$1.92*10^{-63}$	0.54	$2.76*10^{-19}$
A2902	0.90	$4.98*10^{-73}$	0.89	$4.45*10^{-68}$	0.48	$1.09*10^{-12}$
A3101	0.91	$5.62*10^{-71}$	0.91	$3.96*10^{-73}$	0.43	$6.89*10^{-10}$
A6801	0.95	$2.92*10^{-116}$	0.95	$1.64*10^{-118}$	0.42	$1.57*10^{-11}$
A6802	0.83	$9.97*10^{-43}$	0.84	$4.64*10^{-45}$	0.37	$9.02*10^{-7}$
B0702	0.91	$1.73*10^{-189}$	0.91	$2.21*10^{-188}$	0.41	$4.08*10^{-21}$
B0801	0.91	$1.66*10^{-111}$	0.80	$1.47*10^{-65}$	0.75	$6.22*10^{-54}$
B1501	0.85	$1.02*10^{-90}$	0.86	$2.28*10^{-93}$	0.39	$1.09*10^{-12}$
B1801	0.96	$4.44*10^{-141}$	0.96	$5.67*10^{-142}$	0.39	$3.36*10^{-11}$
B2705	0.94	$5.68*10^{-153}$	0.94	$3.13*10^{-153}$	0.35	$2.73*10^{-11}$
B3501	0.85	$1.19*10^{-73}$	0.85	$1.92*10^{-73}$	0.39	$6.41*10^{-11}$
B3901	0.86	$1.17*10^{-46}$	0.85	$2.71*10^{-46}$	0.49	$4.46*10^{-11}$
B4001	0.91	$2.19*10^{-75}$	0.92	$7.44*10^{-80}$	0.42	$9.71*10^{-10}$
B4002	0.89	$1.53*10^{-42}$	0.90	$1.35*10^{-44}$	0.24	$9.67*10^{-3}$
B4403	0.87	$1.04*10^{-85}$	0.88	$5.16*10^{-89}$	0.25	$2.57*10^{-5}$
B5101	0.87	$2.88*10^{-82}$	0.87	$1.11*10^{-80}$	0.32	$7.39*10^{-8}$
B5701	0.88	$1.18*10^{-117}$	0.88	$5.12*10^{-119}$	0.36	$3.22*10^{-12}$
C0304	0.94	$2.93*10^{-102}$	0.94	$2.88*10^{-103}$	0.45	$2.23*10^{-12}$
C0501	0.96	$4.14*10^{-119}$	0.95	$1.29*10^{-117}$	0.48	$5.30*10^{-14}$
C1601	0.90	$1.39*10^{-77}$	0.91	$3.63*10^{-79}$	0.53	$1.71*10^{-16}$

Supplementary Table S4. Independent dataset of non-APE-Gen modeled structures - 16 peptides of the HLA-A0201 receptor[50]. Note for the five decoy peptides were modeled using Docktope tool[54], as they are not found in the PDB.

	Peptide	Method	Affinity (nM)	PDB codes
strong binders	ALWGFFPVL	purified MHC/competitive/radioactivity	2.7	1B0G, 1LP9, 2UWE, 2JCC, 2J8U
	FLPSDFFPSV	cellular MHC/competitive/radioactivity	0.57	1HHH, 3OX8, 3OXR, 3OXS
	LLFGYPVYV	purified MHC/competitive/radioactivity	3.8	1HHK, 1IM3, 2AV7, 2AV1, 1DUZ, 1AO7, 1BD2, 3IXA, 4E5X, 4FTV, 5IRO
medium binders	CINGVCWTV	purified MHC/competitive/radioactivity	55	3MRG
	ILKEPVHGV	purified MHC/competitive/radioactivity	192.3	1HHJ, 1P7Q, 2X4U, 1AKJ
	VLRDDLLEA	purified MHC/competitive/fluorescence	365	3FT4
	AAGIGILTV	purified MHC/competitive/radioactivity	395	2GUO, 2GUO, 3QEQ, 3QDJ, 3QFD
weak binders	RGPGRAFVTI	purified MHC/competitive/radioactivity	4600	3ECB, 3DMM, 1QO3, 1BII, 1DDH, 5IVX
	SLLMWITQC	purified MHC/competitive/radioactivity	21070	1S9W, 2PYE, 2P5E, 2P5W, 2F54, 2F54, 2F53, 2BNR
	RQISQDVKL	purified MHC/competitive/radioactivity	1925	4NO5, 4NO5
	EAAGIGILTV	purified MHC/competitive/fluorescence	14560	2GT9, 4QOK
non-binders	AAEQRRSTI	cellular MHC/competitive/fluorescence	>70000	Docktope model
	DAKRNSKSL	cellular MHC/competitive/fluorescence	>70000	Docktope model
	EIDVSEVKT	cellular MHC/competitive/fluorescence	>70000	Docktope model
	ATKRYPGVM	cellular MHC/competitive/fluorescence	>70000	Docktope model
	ETLNEYKQL	cellular MHC/competitive/fluorescence	>70000	Docktope model

Supplementary Table S5. Overview of the investigated scoring functions.

scoring function	methodology
AutoDock4[38]	empirical/forcefield
Vina[39]	empirical
Vinardo[40]	empirical
Foldx[42]	empirical/forcefield
GradDock[31]	pMHC trained ref2015
DOPE[41]	knowledge-based
3pHLA-score	pHLA trained ref2015

Supplementary Table S6. Pearson's correlation coefficient and corresponding two-sided p-values for different scoring functions evaluated on Dataset 3.

	r	p-value
DOPE[41]	0.62	$7.78 * 10^{-10}$
3pHLA-score	0.56	$5.43 * 10^{-8}$
Vina[39]	0.46	$1.49 * 10^{-5}$
Vinardo[40]	0.43	$6.12 * 10^{-5}$
FoldX[42]	0.25	0.02
AutoDock4[38]	0.18	0.11
GradDock[31]	0.17	0.13

Supplementary Table S7. Per-allele AUROC values achieved by different scoring functions in the virtual screening experiment. Best performing value in a row is bolded.

Allele	3pHLA-score	Vina	Vinardo	AutoDock4	DOPE	FoldX	GradDock
A0101	**0.997859**	0.953229	0.979900	0.845758	0.843517	0.844652	0.851253
A0201	**0.977805**	0.860607	0.914830	0.771790	0.893805	0.763165	0.877715
A0301	**0.983478**	0.872490	0.907125	0.767695	0.670895	0.719420	0.806323
A1101	**0.986580**	0.799687	0.819128	0.595910	0.569345	0.613045	0.747380
A2402	**0.991947**	0.948178	0.977718	0.850310	0.943699	0.738318	0.888586
A2902	**0.986024**	0.939271	0.958358	0.878182	0.921928	0.715233	0.856219
B0702	**0.984132**	0.910110	0.887766	0.701091	0.730810	0.757989	0.824495
B0801	**0.979995**	0.799905	0.895650	0.720445	0.817349	0.676633	0.787534
B1501	**0.968293**	0.841742	0.888293	0.673023	0.739590	0.686241	0.727943
B2705	**0.983617**	0.721595	0.822185	0.692447	0.706670	0.622695	0.721730
B3501	**0.954505**	0.931365	0.908800	0.796937	0.807605	0.710998	0.774555
B4001	**0.993772**	0.872666	0.902276	0.721731	0.654362	0.622001	0.690133
B4002	**0.976913**	0.887100	0.912087	0.762341	0.743367	0.538744	0.711106
B4403	**0.993922**	0.878464	0.933182	0.782636	0.644102	0.534307	0.645015
B5101	**0.964695**	0.942995	0.947765	0.817850	0.841995	0.941030	0.871555
B5701	**0.981696**	0.935688	0.949525	0.836808	0.831701	0.702084	0.734085

Supplementary Table S8. Per-allele AUPRC values achieved by different scoring functions in the virtual screening experiment. Best performing value in a row is bolded.

Allele	3pHLA-score	Vina	Vinardo	AutoDock4	DOPE	FoldX	GradDock
A0101	**0.956977**	0.523399	0.690218	0.192611	0.181234	0.337905	0.290420
A0201	**0.715660**	0.205126	0.298295	0.142011	0.285571	0.183424	0.357065
A0301	**0.740916**	0.277324	0.412307	0.202703	0.091861	0.146112	0.290163
A1101	**0.774023**	0.157361	0.189710	0.072240	0.055838	0.073304	0.140114
A2402	**0.919628**	0.422715	0.670886	0.206803	0.397063	0.132031	0.368762
A2902	**0.861374**	0.523034	0.598588	0.347429	0.348952	0.180041	0.306128
B0702	**0.695089**	0.376450	0.346057	0.091574	0.104016	0.197584	0.176701
B0801	**0.668274**	0.140807	0.354462	0.100157	0.158445	0.110397	0.164774
B1501	**0.800140**	0.257857	0.372958	0.099685	0.093826	0.139662	0.139493
B2705	**0.804437**	0.105399	0.186082	0.100384	0.087655	0.064670	0.168544
B3501	**0.699721**	0.443929	0.335562	0.154223	0.148952	0.146906	0.171589
B4001	**0.896198**	0.214475	0.256000	0.100702	0.068339	0.081446	0.091538
B4002	**0.761602**	0.241524	0.324725	0.135011	0.091169	0.066542	0.091808
B4403	**0.854348**	0.320935	0.469818	0.167838	0.069099	0.066236	0.077369
B5101	**0.795268**	0.531897	0.538648	0.181736	0.195025	0.636270	0.318873
B5701	**0.835307**	0.480516	0.550943	0.237850	0.181619	0.130319	0.115888

Alternative ML regression techniques

In the subsection *Machine learning models* of the *Methods* section we describe how we used Random Forest Regression models to train standard-pHLA-score and 3pHLA-score. It is possible to create variants of standard-pHLA-score and 3pHLA-score using the same protocol but replacing the Random Forest Regression with any other machine learning regression technique. Here we showcase the performance of alternative regression techniques: Linear Regression (LR), Support Vector Machine (SVM) Regression, Partial Least Squares (PLS) Regression and Random Forest (RF) Regression. We used the same dataset (training portion of Dataset 1) to train the models. We extracted the standard features and used them as input to train standard-pHLA-score. We extracted the per-peptide-position features and used them as input to train 3pHLA-score. We performed hyperparameter tuning for each regression model using 5-fold cross-validation and evaluated the performance of the models on the test portion of Dataset 1.

We report the Pearson's correlation between the experimental binding affinities and affinities predicted with standard-pHLA-score (Table S9, Figure S7) and 3pHLA-score (Table S10, Figure S7) trained using different regression techniques. For all regression techniques across all alleles we observe the same pattern as reported for the RF models in the main text: using per-peptide-position features as input to the models increases the performance of the models. RF has overall best performance across alleles out of all regression methods we used (both for standard featurization and per-peptide-position featurization).

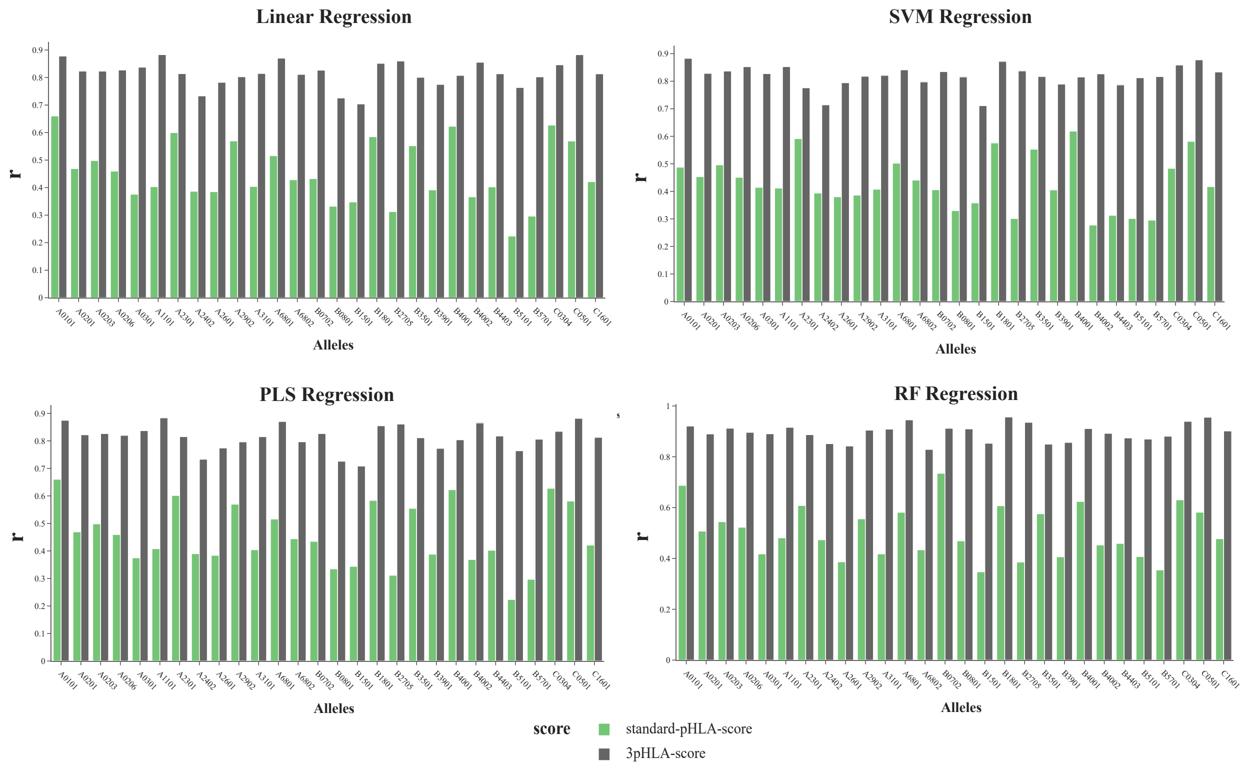

Figure S7. The standard-pHLA-score, and 3pHLA-score are trained using different ML regression techniques: Linear Regression, Support Vector Machine (SVM) Regression, Partial Least Squares (PLS) Regression and Random Forest (RF) Regression. Their performance is evaluated and compared on the test portion of Dataset 1. Results are reported for individual alleles, listed on the x-axis. The regression power of the scores is quantified using Pearson's r, on the y-axis.

Supplementary Table S9. Pearson's correlation coefficient and corresponding two-sided p-values between the experimental binding affinities and predicted standard-pHLA-score. The predictors were trained using the standard featurization as input to different machine learning models (linear regression - LR; support vector machine - SVM; partial least squares - PLS; random forest - RF). Models were trained on the training portion of Dataset 1. Results were obtained on the test portion of Dataset 1 and are reported for each allele.

	LR		SVM		PLS		RF	
Allele	r_p	p-value	r_p	p-value	r_p	p-value	r_p	p-value
A0101	0.66	$2.57*10^{-39}$	0.49	$1.63*10^{-19}$	0.66	$2.45*10^{-39}$	0.69	$1.07*10^{-43}$
A0201	0.47	$3.34*10^{-44}$	0.45	$4.61*10^{-41}$	0.47	$3.23*10^{-44}$	0.51	$1.33*10^{-52}$
A0203	0.50	$8.49*10^{-19}$	0.50	$1.27*10^{-18}$	0.50	$8.51*10^{-19}$	0.54	$9.01*10^{-23}$
A0206	0.46	$1.23*10^{-08}$	0.45	$2.55*10^{-08}$	0.46	$1.27*10^{-08}$	0.52	$4.15*10^{-11}$
A0301	0.38	$1.50*10^{-20}$	0.41	$5.47*10^{-25}$	0.37	$2.01*10^{-20}$	0.42	$1.95*10^{-25}$
A1101	0.40	$2.63*10^{-12}$	0.41	$8.25*10^{-13}$	0.41	$1.42*10^{-12}$	0.48	$1.65*10^{-17}$
A2301	0.60	$2.92*10^{-19}$	0.59	$1.18*10^{-18}$	0.60	$2.20*10^{-19}$	0.61	$6.80*10^{-20}$
A2402	0.39	$3.05*10^{-11}$	0.39	$1.2*10^{-11}$	0.39	$2.01*10^{-11}$	0.47	$9.13*10^{-17}$
A2601	0.39	$1.16*10^{-9}$	0.38	$1.97*10^{-9}$	0.38	$1.34*10^{-9}$	0.39	$9.87*10^{-10}$
A2902	0.57	$4.28*10^{-18}$	0.39	$2.66*10^{-8}$	0.57	$4.30*10^{-18}$	0.56	$4.07*10^{-17}$
A3101	0.40	$1.26*10^{-8}$	0.41	$9.29*10^{-9}$	0.40	$1.26*10^{-08}$	0.42	$3.59*10^{-09}$
A6801	0.52	$1.59*10^{-17}$	0.50	$1.5*10^{-16}$	0.52	$1.59*10^{-17}$	0.58	$7.65*10^{-23}$
A6802	0.43	$1.03*10^{-8}$	0.44	$3.53*10^{-9}$	0.44	$2.56*10^{-9}$	0.43	$6.39*10^{-9}$
B0702	0.43	$1.35*10^{-23}$	0.41	$1.09*10^{-20}$	0.43	$8.74*10^{-24}$	0.73	$1.16*10^{-83}$
B0801	0.33	$7.01*10^{-9}$	0.33	$9.17*10^{-9}$	0.33	$5.49*10^{-9}$	0.47	$3.26*10^{-17}$
B1501	0.35	$2.02*10^{-10}$	0.36	$5.57*10^{-11}$	0.34	$3.38*10^{-10}$	0.35	$2.19*10^{-10}$
B1801	0.58	$1.91*10^{-25}$	0.58	$1.59*10^{-24}$	0.58	$2.62*10^{-25}$	0.61	$8.41*10^{-28}$
B2705	0.31	$4.57*10^{-9}$	0.30	$1.77*10^{-08}$	0.31	$5.38*10^{-9}$	0.39	$2.32*10^{-13}$
B3501	0.55	$3.79*10^{-22}$	0.55	$3.18*10^{-22}$	0.55	$2.28*10^{-22}$	0.58	$2.35*10^{-24}$
B3901	0.39	$3.55*10^{-7}$	0.41	$1.28*10^{-7}$	0.39	$4.66*10^{-7}$	0.41	$1.17*10^{-7}$
B4001	0.62	$4.02*10^{-22}$	0.62	$8.91*10^{-22}$	0.62	$4.31*10^{-22}$	0.62	$3.12*10^{-22}$
B4002	0.37	$3.82*10^{-5}$	0.28	$2.14*10^{-3}$	0.37	$3.42*10^{-5}$	0.45	$2.04*10^{-7}$
B4403	0.40	$6.55*10^{-12}$	0.31	$1.63*10^{-7}$	0.40	$6.85*10^{-12}$	0.46	$2.00*10^{-15}$
B5101	0.22	$2.42*10^{-4}$	0.30	$6.02*10^{-7}$	0.22	$2.53*10^{-4}$	0.41	$5.67*10^{-12}$
B5701	0.30	$1.08*10^{-8}$	0.30	$1.21*10^{-8}$	0.30	$1.03*10^{-8}$	0.35	$4.61*10^{-12}$
C0304	0.63	$3.09*10^{-25}$	0.48	$3.62*10^{-14}$	0.63	$2.89*10^{-25}$	0.63	$1.45*10^{-25}$
C0501	0.57	$1.46*10^{-20}$	0.58	$1.44*10^{-21}$	0.58	$1.50*10^{-21}$	0.58	$1.36*10^{-21}$
C1601	0.42	$1.85*10^{-10}$	0.42	$3.03*10^{-10}$	0.42	$1.97*10^{-10}$	0.48	$2.31*10^{-13}$

Supplementary Table S10. Pearson's correlation coefficient and corresponding two-sided p-values between the experimental binding affinities and predicted 3pHLA-score. The predictors were trained using the per-peptide-position featurization as input to different machine learning models (linear regression - LR; support vector machine - SVM; partial least squares - PLS; random forest - RF). Models were trained on the training portion of Dataset 1. Results were obtained on the test portion of Dataset 1 and are reported for each allele.

	LR		SVM		PLS		RF	
Allele	r_p	p-value	r_p	p-value	r_p	p-value	r_p	p-value
A0101	0.88	$2.91*10^{-98}$	0.87	$8.99*10^{-96}$	0.87	$1.03*10^{-96}$	0.92	$4.18*10^{-125}$
A0201	0.82	$8.53*10^{-195}$	0.82	$9.38*10^{-195}$	0.82	$9.39*10^{-194}$	0.89	$3.85*10^{-269}$
A0203	0.82	$1.44*10^{-69}$	0.83	$3.57*10^{-70}$	0.83	$1.37*10^{-70}$	0.91	$1.37*10^{-108}$
A0206	0.83	$4.05*10^{-36}$	0.83	$7.10*10^{-36}$	0.82	$5.61*10^{-35}$	0.90	$3.90*10^{-50}$
A0301	0.84	$6.49*10^{-151}$	0.82	$1.44*10^{-139}$	0.84	$1.09*10^{-150}$	0.89	$5.25*10^{-196}$
A1101	0.88	$1.14*10^{-92}$	0.84	$1.17*10^{-75}$	0.88	$6.67*10^{-93}$	0.92	$2.68*10^{-111}$
A2301	0.81	$1.44*10^{-44}$	0.76	$4.05*10^{-36}$	0.82	$8.10*10^{-45}$	0.89	$1.36*10^{-62}$
A2402	0.73	$1.21*10^{-47}$	0.70	$4.35*10^{-41}$	0.73	$1.23*10^{-47}$	0.85	$1.59*10^{-78}$
A2601	0.78	$1.99*10^{-49}$	0.77	$1.61*10^{-47}$	0.77	$9.13*10^{-48}$	0.84	$5.92*10^{-64}$
A2902	0.80	$5.50*10^{-45}$	0.80	$6.60*10^{-44}$	0.80	$8.86*10^{-44}$	0.90	$4.98*10^{-73}$
A3101	0.81	$6.32*10^{-45}$	0.80	$1.30*10^{-42}$	0.82	$4.88*10^{-45}$	0.91	$5.62*10^{-71}$
A6801	0.87	$3.11*10^{-74}$	0.82	$6.25*10^{-60}$	0.87	$2.95*10^{-74}$	0.95	$2.92*10^{-116}$
A6802	0.81	$1.32*10^{-39}$	0.77	$5.80*10^{-34}$	0.80	$2.95*10^{-37}$	0.83	$9.97*10^{-43}$
B0702	0.83	$6.63*10^{-123}$	0.82	$7.27*10^{-122}$	0.83	$7.30*10^{-123}$	0.91	$1.73*10^{-189}$
B0801	0.73	$1.59*10^{-48}$	0.80	$1.64*10^{-66}$	0.73	$1.42*10^{-48}$	0.91	$1.66*10^{-111}$
B1501	0.70	$1.38*10^{-48}$	0.69	$1.40*10^{-46}$	0.71	$2.03*10^{-49}$	0.85	$1.02*10^{-90}$
B1801	0.85	$7.44*10^{-75}$	0.86	$1.82*10^{-77}$	0.85	$4.71*10^{-76}$	0.96	$4.44*10^{-141}$
B2705	0.86	$1.43*10^{-99}$	0.82	$1.28*10^{-83}$	0.86	$4.02*10^{-100}$	0.94	$5.68*10^{-153}$
B3501	0.80	$2.38*10^{-59}$	0.80	$1.17*10^{-59}$	0.81	$4.36*10^{-62}$	0.85	$1.19*10^{-73}$
B3901	0.77	$6.88*10^{-33}$	0.75	$2.42*10^{-30}$	0.77	$1.43*10^{-32}$	0.86	$1.17*10^{-46}$
B4001	0.81	$1.10*10^{-45}$	0.80	$1.06*10^{-43}$	0.80	$5.72*10^{-45}$	0.91	$2.19*10^{-75}$
B4002	0.86	$1.69*10^{-35}$	0.78	$1.72*10^{-25}$	0.87	$3.82*10^{-37}$	0.89	$1.53*10^{-42}$
B4403	0.81	$8.04*10^{-65}$	0.76	$7.25*10^{-53}$	0.82	$5.64*10^{-66}$	0.87	$1.04*10^{-85}$
B5101	0.76	$1.08*10^{-51}$	0.80	$1.44*10^{-60}$	0.76	$8.67*10^{-52}$	0.87	$2.88*10^{-82}$
B5701	0.80	$7.98*10^{-82}$	0.81	$5.92*10^{-83}$	0.81	$4.19*10^{-83}$	0.88	$1.18*10^{-117}$
C0304	0.85	$6.17*10^{-61}$	0.84	$3.40*10^{-60}$	0.83	$9.60*10^{-58}$	0.94	$2.93*10^{-102}$
C0501	0.88	$1.73*10^{-74}$	0.87	$1.03*10^{-68}$	0.88	$4.97*10^{-74}$	0.96	$4.14*10^{-119}$
C1601	0.81	$7.69*10^{-51}$	0.82	$1.20*10^{-52}$	0.81	$1.06*10^{-50}$	0.90	$1.39*10^{-77}$

3pHLA-score comparison with sequence-based approaches in the epitope discovery setting

We developed the 3pHLA-score with the purpose of structure-based virtual screening. In that context, we have compared its performance mainly to structure-based scoring functions. However, as we mention in the introduction, current methods for scoring peptide HLAs are mostly sequence-based. Sequence-based scores do not use structure as input and thus can not be used for structure-based virtual screens. Nevertheless, it is interesting to see how 3pHLA-score compares to the most widely used sequence-based scores. Here we evaluate the performance of 3pHLA-score, MHCFlurry2.0[10] and NetMHCpan4.1[58] in an epitope discovery setting (Dataset 2).

Average AUROC and AUPRC values across all alleles are reported in Table S11 for the compared scoring functions. Figure S8 shows the AUROC and AUPRC curves along with violin plots that depict the distribution of predicted scores across the binder and non-binder peptides from Dataset 2. Finally, Tables S12, S13 show the AUROC and AUPRC obtained for each allele. As expected, sequence-based approaches have very good performance across all alleles with MHCFlurry2.0 having the highest average AUROC and AUPRC (0.993 and 0.865 respectively). 3pHLA-score lags behind the sequence-based approaches with AUROC and AUPRC of 0.977 and 0.712. 3pHLA-score still has comparable performance for most of the alleles in terms of AUROC values (Table S13).

It is important to note that the dataset on which this experiment is performed (Dataset 2) is left out of the training of 3pHLA-score. However, we do not know if MHCFlurry2.0 or NetMHCpan4.1 have had a part of this dataset in their training, which might give them a slight advantage. Structure-based scoring functions are inherently more difficult to train. To the best of our knowledge structure-based scoring functions have not yet come close to the performance of sequence-based methods. The comparable performance of 3pHLA-score shows promise that structure-based approached can reach the accuracies of sequence-based approaches and can bridge the gaps that we mention in the introduction.

Supplementary Table S11. AUROC and AUPRC values aggregated for the virtual screening experiment across HLA alleles. The highest values are bolded.

	AUROC	AUPRC
3pHLA-score	0.977	0.712
MHCFlurry2.0[10]	**0.993**	**0.865**
NetMHCpan4.1[58]	0.991	0.855

Supplementary Table S12. Per-allele AUPRC values achieved by two sequence-based scoring functions and 3pHLA-score in the virtual screening experiment. The best performance in each row is bolded.

Allele	3pHLA-score	MHCFlurry2.0	NetMHCpan4.1
A0101	0.956977	0.948338	**0.967984**
A0201	0.715660	0.811894	**0.823205**
A0301	0.740916	**0.859079**	0.832935
A1101	0.774023	**0.917182**	0.905915
A2402	0.919628	**0.961677**	0.922089
A2902	0.861373	**0.919257**	0.883255
B0702	0.695089	**0.940622**	0.931435
B0801	0.668274	**0.949789**	0.901494
B1501	0.800140	**0.883350**	0.807565
B2705	0.804437	**0.950496**	0.793621
B3501	0.699721	**0.890328**	0.862872
B4001	0.896198	**0.907023**	0.883645
B4002	0.761602	**0.834503**	0.786427
B4403	0.854348	**0.904832**	0.877956
B5101	0.795268	**0.935606**	0.875531
B5701	0.835306	**0.913895**	0.888805

Supplementary Table S13. Per-allele AUROC values achieved by two sequence-based scoring functions and 3pHLA-score in the virtual screening experiment. The best performance in each row is bolded.

Allele	3pHLA-score	MHCFlurry2.0	NetMHCpan4.1
A0101	0.997860	0.998440	**0.998790**
A0201	0.977805	0.985580	**0.986395**
A0301	0.983478	**0.994025**	0.988960
A1101	0.986580	**0.995910**	0.995730
A2402	0.991955	**0.998285**	0.997485
A2902	0.986033	**0.995858**	0.994907
B0702	0.984140	**0.997170**	0.997115
B0801	0.980005	**0.996925**	0.995970
B1501	0.968315	**0.994275**	0.990650
B2705	0.983617	**0.997285**	0.991645
B3501	0.954505	**0.987875**	0.983180
B4001	0.993775	**0.997815**	0.996590
B4002	0.976924	**0.988520**	0.984520
B4403	0.993925	**0.995845**	0.994845
B5101	0.964695	**0.991275**	0.986595
B5701	0.981700	**0.995110**	0.993540

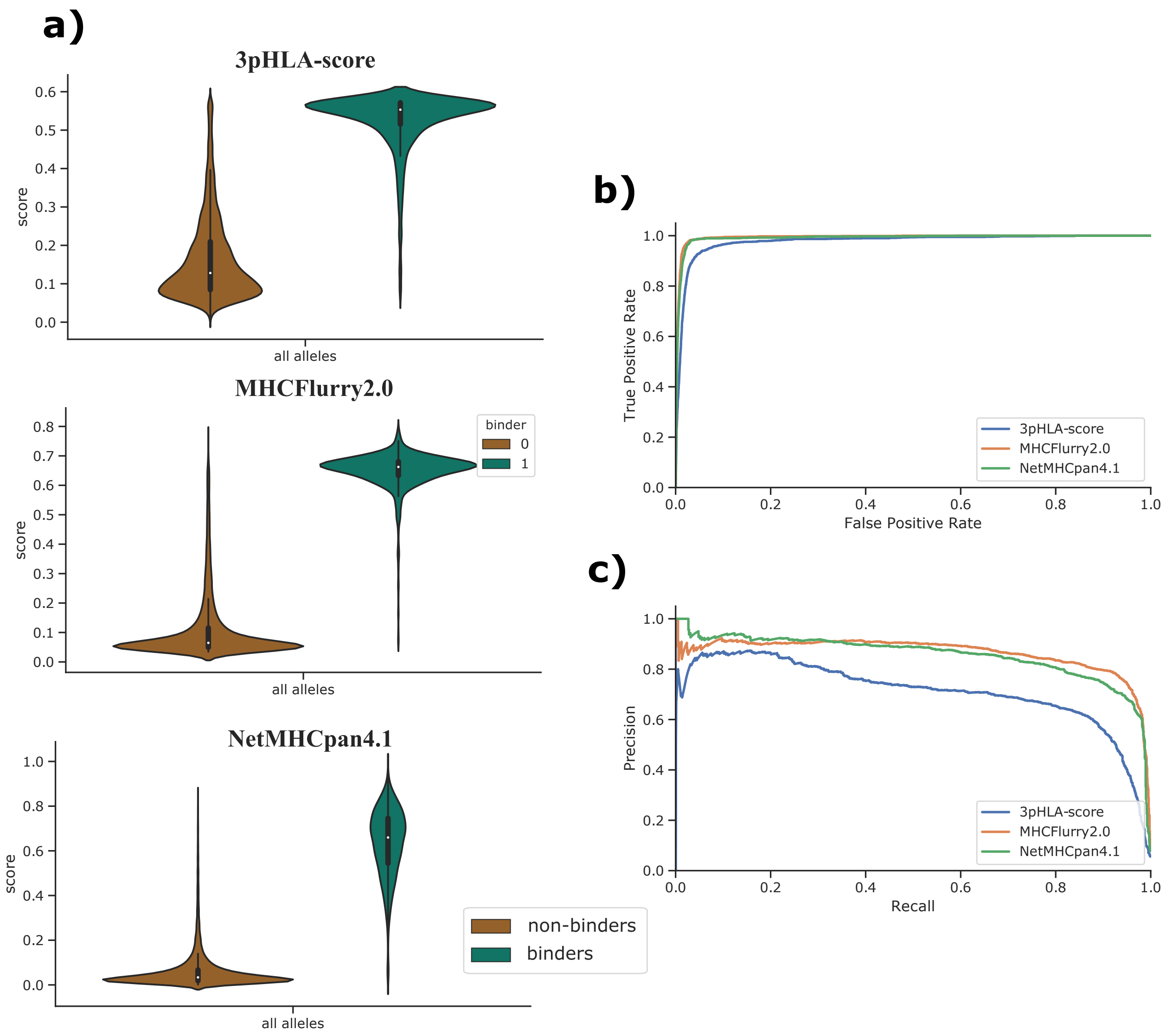

Figure S8. Comparing 3pHLA-score to sequence-based approached (MHCFlurry2.0, NetMHCpan4.1) in the virtual screening setting. Results are aggregated across alleles. a) Violin plots show the distribution of predicted binding affinities for binders (green) and non-binders (brown) and give an estimate of how well different scoring functions distinguish binders from non-binders in this setting. b) ROC-curves for different scoring functions in the virtual screening setting; c) PR-curves in the virtual screening setting.
